# m^6^A-induced lncRNA RP11 triggers the dissemination of colorectal cancer cells via upregulation of Zeb1

**DOI:** 10.1186/s12943-019-1014-2

**Published:** 2019-04-13

**Authors:** Yingmin Wu, Xiangling Yang, Zhuojia Chen, Lin Tian, Guanmin Jiang, Feng Chen, Jiexin Li, Panpan An, Linlin Lu, Nan Luo, Jun Du, Hong Shan, Huanliang Liu, Hongsheng Wang

**Affiliations:** 1 Guangdong Key Laboratory of Chiral Molecule and Drug Discovery, and Guangdong Provincial Key Laboratory of New Drug Design and Evaluation, School of Pharmaceutical Sciences, Sun Yat-sen University, Guangzhou, Guangdong, 510006 China; 2 Guangdong Provincial Key Laboratory of Colorectal and Pelvic Floor Diseases, Guangdong Institute of Gastroenterology, The Sixth Affiliated Hospital, Sun Yat-sen University, Guangzhou, Guangdong, 510655 China; 3 Sun Yat-sen University Cancer Center; State Key Laboratory of Oncology in South China; Collaborative Innovation Center for Cancer Medicine, Guangzhou, 510060 Guangdong China; 4 Department of Pharmacy, The Fifth Affiliated Hospital of Sun Yat-sen University, Zhuhai, 519000 Guangdong China; 5 Department of Clinical Laboratory, The Fifth Affiliated Hospital, Sun Yat-sen University, Zhuhai, 519000 Guangdong China; 6 Key Laboratory of Biomedical Imaging of Guangdong Province, Guangdong Provincial Engineering Research Center of Molecular Imaging, The Fifth Affiliated Hospital of Sun Yat-sen University, Zhuhai, 519000 Guangdong China; 7 Department of Clinical Laboratory, The Sixth Affiliated Hospital, Sun Yat-sen University, Guangzhou, 510655 Guangdong China

**Keywords:** LncRNA RP11, CRC, Zeb1, m^6^A, hnRNPA2B1, Cell dissemination

## Abstract

**Background:**

Long noncoding RNAs (lncRNAs) have emerged as critical players in cancer progression, but their functions in colorectal cancer (CRC) metastasis have not been systematically clarified.

**Methods:**

lncRNA expression profiles in matched normal and CRC tissue were checked using microarray analysis. The biological roles of a novel lncRNA, namely RP11-138 J23.1 (RP11), in development of CRC were checked both in vitro and in vivo. Its association with clinical progression of CRC was further analyzed.

**Results:**

RP11 was highly expressed in CRC tissues, and its expression increased with CRC stage in patients. RP11 positively regulated the migration, invasion and epithelial mesenchymal transition (EMT) of CRC cells in vitro and enhanced liver metastasis in vivo. Post-translational upregulation of Zeb1, an EMT-related transcription factor, was essential for RP11-induced cell dissemination. Mechanistically, the RP11/hnRNPA2B1/mRNA complex accelerated the mRNA degradation of two E3 ligases, Siah1 and Fbxo45, and subsequently prevented the proteasomal degradation of Zeb1. m^6^A methylation was involved in the upregulation of RP11 by increasing its nuclear accumulation. Clinical analysis showed that m^6^A can regulate the expression of RP11, further, RP11 regulated Siah1-Fbxo45/Zeb1 was involved in the development of CRC.

**Conclusions:**

m^6^A-induced lncRNA RP11 can trigger the dissemination of CRC cells via post-translational upregulation of Zeb1. Considering the high and specific levels of RP11 in CRC tissues, our present study paves the way for further investigations of RP11 as a predictive biomarker or therapeutic target for CRC.

**Electronic supplementary material:**

The online version of this article (10.1186/s12943-019-1014-2) contains supplementary material, which is available to authorized users.

## Introduction

Colorectal cancer (CRC), also known as large bowel cancer, is a major public health problem worldwide [1]. Epidemiological data have revealed that the 5-year survival rate of CRC patients ranges from 90% for patients with stage I disease to 10% for those with metastatic disease [2]. Although numerous studies have revealed that alterations in oncogenes and tumour suppressor genes contribute to tumorigenesis and the development of CRC [3], the precise molecular mechanisms underlying CRC pathogenesis, particularly for metastasis, remain to be fully elucidated.

Long noncoding RNAs (lncRNAs), which are more than 200 nt in length and have limited or no protein-coding capacity, play both oncogenic and tumour suppressor roles in tumorigenesis and progression [4, 5]. LncRNAs can regulate gene expression via multiple mechanisms, including chromatin remodelling, modulation of the activity of transcriptional regulators, and posttranscriptional modifications [5]. Dysregulated lncRNA expression has been reported to modulate the progression of various types of cancers, such as bladder, prostate, lung, breast, gastric and colorectal cancers [6, 7]. Increasing evidence suggests that lncRNAs can trigger metastatic progression, increase chromosomal instability, and promote CRC tumorigenesis [8–10]. Therefore, further identification of CRC-related lncRNAs and investigations of their functions in CRC are imperative.

Metastasis is the major cause of CRC related death [11]. The epithelial mesenchymal transition (EMT), a process by which epithelial cells gain a migratory and invasive mesenchymal phenotype [12], is considered as the first and most important step for cancer cell metastasis. During EMT, epithelial cells can acquire mesenchymal components and motility features, lose epithelial components and cell adhesion, and infiltrate into the tumour vasculature [13]. Increasing evidences indicate that EMT is a pivotal step for tumour infiltration and distant metastasis in a variety of carcinomas [14]. EMT-transcription factors (EMT-TFs), including Twist, Snail, and Zeb1, have been implicated in the control of EMT [15]. The important role of Zeb1 in EMT regulation has been described for many cancer types [16, 17]. LncRNAs have been reported to regulate EMT-TFs and subsequently trigger the EMT of cancer cells [18]. We are interested in determining whether any lncRNAs exist that can regulate EMT-TFs to trigger the EMT and dissemination of CRC cells.

In this study, a CRC-associated lncRNA (RP11, RP11-138 J23.1) that displayed a remarkable trend towards increasing expression levels from normal colorectal to CRC tissues was identified and selected for further validation and functional analysis in terms of CRC progression. We demonstrated that post-translational upregulation of Zeb1 is required for the lncRNA RP11-induced EMT and dissemination of CRC cells.

## Materials and methods

### Microarray and computational analysis

Fresh paired normal and histologically confirmed CRC tumour tissues were obtained from 3 stage I CRC cases and 3 stage IV cases with distant metastasis before any treatment during surgery from the Sixth Affiliated Hospital of Sun Yat-sen University from February to October 2014. Total RNA from the samples (3 stage I CRC tissues, 3 stage IV CRC tissues, and their corresponding paired nontumour tissues) was extracted, amplified and transcribed into fluorescent cRNA using the Quick Amp Labeling kit (Agilent Technologies, Palo Alto, CA, USA). The labelled cRNA was then hybridized onto the Human LncRNA Array v2.0 (8 × 60 K, ArrayStar, Rockville, MD, USA), and after the washing steps, the arrays were scanned with the Agilent Scanner G2505B. Agilent Feature Extraction software (version 10.7.3.1) was used to analyze the acquired array images. Quantile normalization and subsequent data processing were performed using the GeneSpring GX v11.5.1 software package (Agilent Technologies). The differentially expressed lncRNAs with statistical significance were identified using Volcano Plot Filtering. The threshold used to screen upregulated or downregulated lncRNAs was a fold change ≥2.0 and *p* < 0.05.

### Database (DB) search

The expression of lncRNA RP11 in CRC and other cancers was analyzed using the GEPIA (Gene Expression Profiling Interactive Analysis) online database (http://gepia.cancer-pku.cn). The expression of RP11 between tumour and normal tissues or among different stages of CRC was also analyzed with GEPIA. GEPIA can deliver fast and customizable functionalities based on data from The Cancer Genome Atlas (TCGA) and provide key interactive and customizable functions, including differential expression analysis, correlation analysis and patient survival analysis [19]. We used the Kaplan-Meier plotter to assess the prognostic value of RP11, Zeb1, and their normalization to Siah1 or Fbxo45 expression in CRC patients based on the data from the GEPIA online database. The high expression was defined as greater than the median of the values of transcripts, while the low expression was defined as less than the median of the values of transcripts.

Data about the expression of Zeb1 in CRC and normal tissues were further obtained from the Oncomine database (www.oncomine.org) as follows: Hong Colorectal [20] and Skrzypczak colorectal 2 [21]. The sample information and expression data are available in the Gene Expression Omnibus (GEO) database [Accession nos. GSE2091 (Skrzypczak colorectal 2) and GSE9348 (Hong Colorectal) at www.ncbi.nlm.nih.gov/geo].

The expression profiles of Zeb1, Fbxo45, METTL3 and Siah1 among the N stages of CRC in patients were downloaded from LinkedOmics (http://www.linkedomics.org), which is a publicly available portal that includes multi-omics data from all 32 cancer types from TCGA. The LinkedOmics website allowed a flexible exploration of associations between a molecular or clinical attribute of interest and all other attributes, providing the opportunity to analyse and visualize associations between billions of attribute pairs for each cancer cohort [22].

### Animal studies

All animal experiments were complied with the Zhongshan School of Medicine Policy on the Care and Use of Laboratory Animals. To evaluate the potential roles of RP11 in the growth of CRC, ten female BALB/c nude mice (4 weeks old) purchased from Sun Yat-sen University (Guangzhou, China) Animal Center were raised under pathogen-free conditions and randomly divided into two groups. HCT-15 RP11 stable overexpression or control cells (2 × 10^6^ per mouse) diluted in 100 μl normal medium + 100 μl Matrigel (BD Biosciences) were subcutaneously injected into immunodeficient mice to investigate tumour growth. When the tumours of all mice grew into visible tumours, the tumour volumes were measured every 3 d using manual callipers and calculated using the formula V = 1/2 × larger diameter × (smaller diameter) ^2^. At the end of the experiment, mice were sacrificed, and tumours were removed and weighed for use in histological and other analyses.

For the in vivo liver metastasis model, HCT-15 RP11 stable overexpression or control cells (1 × 10^6^ per mouse) were injected into both male and female BALB/c nude mice (*n* = 7 for each group) via the tail vein to analyze distant metastasis. Eight weeks after injection, the experiment was terminated, and livers were analyzed for the presence of metastatic tumours.

### Protein stability

To measure protein stability, cells were treated with cycloheximide (CHX, final concentration 100 μg/ml) for the indicated time periods. Zeb1 expression was measured by western blot analysis.

### RNA immunoprecipitation

RNA immunoprecipitation (RIP) experiments were performed using a Magna RIP RNA-Binding Protein Immunoprecipitation Kit (Millipore, Bedford, MA, USA) according to previously described procedures [23]. Antibodies for RIP assays of IgG, Zeb1, Siah1, Fbxo45, hnRNPA2B1, and m^6^A were diluted 1: 1000. After RIP, RNA concentrations were measured using the Qubit® RNA High-sensitivity (HS) Assay Kit and Qubit 2.0. The co-precipitated RNAs were detected by reverse transcription (RT)-PCR. The gene-specific primers used for detecting RP11 were presented in Additional file 2 :Table S2. RNA expression was normalized to the total amount of RNA used for reverse transcription.

### RNA pull-down/mass spectroscopy analysis

LncRNA-RP11 and its antisense RNA were transcribed in vitro from the pGEM-T-RP11 vector, biotin-labelled with the Biotin RNA Labeling Mix (Roche Diagnostics, Indianapolis, IN, USA) and T7/SP6 RNA polymerase (Roche), treated with RNase-free DNase I (Roche), and purified with an RNeasy Mini Kit (Qiagen, Valencia, CA, USA). One milligram of protein from the extracts of HCT-15 cells stably transfected with pcDNA3.1-RP11 was then mixed with 50 pmoles of biotinylated RNA, incubated with streptavidin agarose beads (Invitrogen, Carlsbad, CA, USA), and washed. The proteins were resolved by sodium dodecyl sulfate-polyacrylamide gel electrophoresis (SDS-PAGE) and silver-stained, and the specific bands were excised. In-gel proteolysis was performed using trypsin (89,871, Pierce, Rockford, IL, USA). Mass spectroscopy (MS) analysis was then performed on a MALDI-TOF instrument (Bruker Daltonics) as described elsewhere [24].

### mRNA stability

To measure RNA stability in HCT-15 RP11 stable overexpression or control cells, 5 μg/ml actinomycin D (Act-D, Catalogue #A9415, Sigma, St. Louis, MO, USA) was added to cells. After incubation at the indicated times, cells were collected, and RNA was isolated for qRT-PCR. The mRNA half-life (t1/2) of *ZEB1*, *Siah1* or *Fbxo45* was calculated using ln2/slope, and GAPDH was used for normalization.

### Statistical analysis

Statistical analysis was performed using SPSS software (SPSS, Chicago, Illinois, USA). The expression levels of lncRNA RP11 in CRC patients were compared with the paired-sample *t* test. Survival curves were generated using the Kaplan-Meier method, and the differences were analysed with the log-rank test. The χ^2^ test, Fisher’s exact probability, and Student’s *t*-test were used for comparisons between groups. Data were expressed as the mean ± standard deviation (SD) from at least three independent experiments. All *P* values were two-sided and obtained using SPSS v. 16.0 software (Chicago, IL, USA). *p* < 0.05 was considered statistically significant.

## Results

### RP11 is upregulated in CRC cells and tissues

**Fig. 1:**
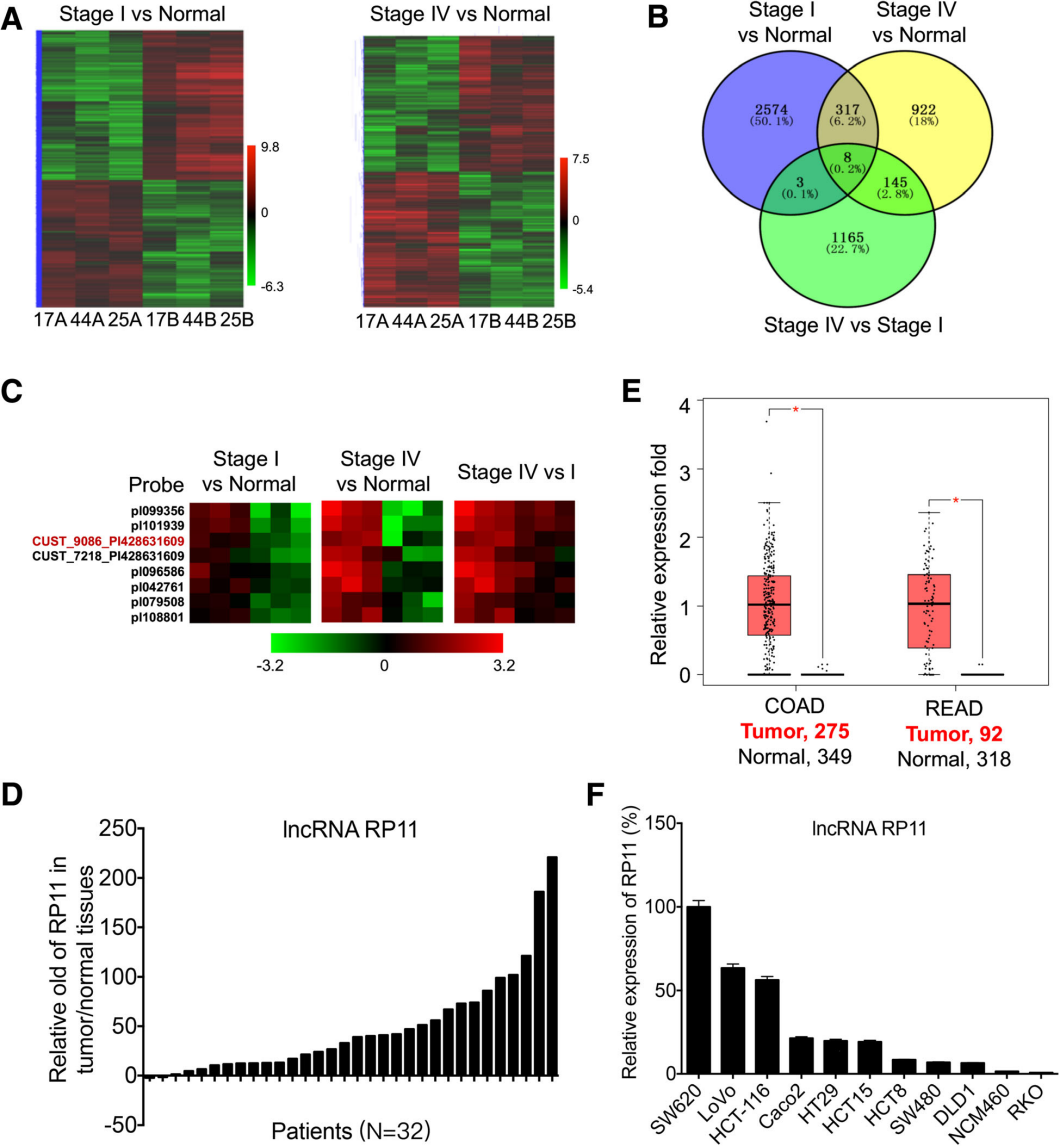
RP11 is increased in CRC cells and tissues. **a** Heat-maps of lncRNAs that were differentially expressed between stage I samples (**a**, cancer tissues) and matched adjacent normal samples (**b**, normal samples) (*left*) or between stage IV samples and matched adjacent normal samples (*right*). The colour scale shown on the left illustrates the relative RNA expression levels; red represents high expression, and green represents low expression. **b** Venn diagram showing the overlapping of 2-fold upregulated lncRNAs between stage I samples and normal samples, stage IV and normal, and stage IV and stage I. **c** Heat-maps of the 8 lncRNAs upregulated simultaneously between stage I samples and normal samples, between stage IV and normal, and between stage IV and stage I (Red probe targeted lncRNA RP11). **d** The relative fold of RP11 in 32 paired human colon cancer tissues versus its matched adjacent normal mucosa tissues. **e** The relative expression of RP11 in colon (*left*) and rectal (*right*) cancer tissues and their corresponding adjacent normal tissues based on data available from TCGA database. **f** The levels of RP11 in CRC cell lines and human colon mucosal epithelial NCM460 cells were measured by qRT-PCR. Data are presented as the mean ± SD from three independent experiments. *p < 0.05 compared with control

To identify potential oncogenic lncRNAs involved in the tumorigenesis and progression of CRC, we analysed lncRNA expression profiles in matched normal and CRC tissue pairs (3 stage I CRC cases and 3 stage IV CRC cases, full data available in the GEO, Accession Number GSE110715) using microarray analysis. Hierarchical clustering showed systematic variations in lncRNA expression between stage I CRC, stage IV CRC, and their corresponding paired adjacent normal samples (Fig. 1 a). The differentially expressed lncRNAs between the CRC tissues and paired adjacent samples were further analysed. As shown in Fig. 1 b, stage I CRC and stage IV CRC tissues shared 325 lncRNAs that were upregulated, with a ≥ 2.0-fold change relative to their corresponding paired nontumour counterparts. Among these lncRNAs, 8 also exhibited greater expression in stage IV CRC tissues than that in stage I tissues (Fig. 1 b&c, Additional file 2 :Table S1). To validate the findings of microarray analysis, we chose the 8 upregulated candidates and randomly selected 2 downregulated lncRNAs to analyse their expression levels by qRT-PCR in 5 pairs of CRC and corresponding nontumour tissues (Additional file 1: Figure S1 A). The results confirmed that all 8 upregulated lncRNAs were overexpressed in CRC, whereas the expression levels of the pl078441 and agiseq14311 target genes were decreased (*p* < 0.05 for all).

Among the eight candidate genes, targets of the CUST_8502_PI428631609 (lncRNA RP11, RP11-138 J23.1) and CUST_9335_PI428631609 (lncRNA AC123023.1) probes have been shown to be lncRNAs. Microarray analysis suggested that the elevation in lncRNA RP11 (RP11) in CRC tissues versus adjacent normal tissues was greater than that of lncRNA AC123023.1 (Table S3). qRT-PCR confirmed that the abundance of RP11 was significantly greater than that of lncRNA AC123023.1 in 5 CRC tissues (Additional file 1: Figure S1 C). RP11 located at Chromosome 5: 104,079,911-104,105,403 with the transcript length 574 nt (ENSG00000251026, Additional file 1: Figure S1 B). It was poly A-tailed due to the enrichment in bound fractions was 11-fold greater than that in unbound fractions by use of polydT-beads pull down and qRT-PCR.

To confirm the role of RP11 in the progression of CRC, we compared RP11 levels in CRC tissues and paired adjacent non-cancerous mucosa from 32 individual patients (Table S1). RP11 was successfully amplified in all tumour and normal specimens analysed. According to the qRT-PCR analysis, RP11 expression was significantly increased in 30 out of 32 (93.8%) tumour samples compared with the adjacent normal mucosa tissues (Fig. 1 d). In this cohort, the average expression level of RP11 in the tumour tissues was 48-fold greater than that in the adjacent normal mucosa tissues. However, there was no significant difference in RP11 expression between different ages, sexes or stages (Table S1), which might be due to the small sample size.

We further assessed RP11 expression in a TCGA pan-cancer dataset obtained from the GEPIA online database (http://gepia.cancer-pku.cn). TCGA data confirmed that the expression of RP11 in colon and rectal carcinoma (COAD, READ) was significantly (*p* < 0.05) greater than that in the adjacent normal tissues (Fig. 1 e). In addition, the expression of RP11 in COAD and READ was relatively high among all measured cancers (Additional file 1: Figure S1 D and E). RP11 expression was verified in multiple colon cancer cell lines, namely, SW620, LoVo, HCT-116, Caco2, HT29, HCT-15, HCT-8, SW480, DLD1, and RKO, and in human colon mucosal epithelial NCM460 cells. The results indicated that the RP11 levels in all of the measured CRC cell lines, except RKO, were greater than that in NCM460 cells (Fig. 1 f). SW620 cells, which were primarily derived from lymph node metastases in CRC patients, had the highest level of RP11 among all analysed cell lines (Fig. 1 f). Collectively, these data show that lncRNA RP11 is increased in CRC cells and tissues.

### RP11 triggers the dissemination of CRC cells both in vitro and in vivo

**Fig. 2:**
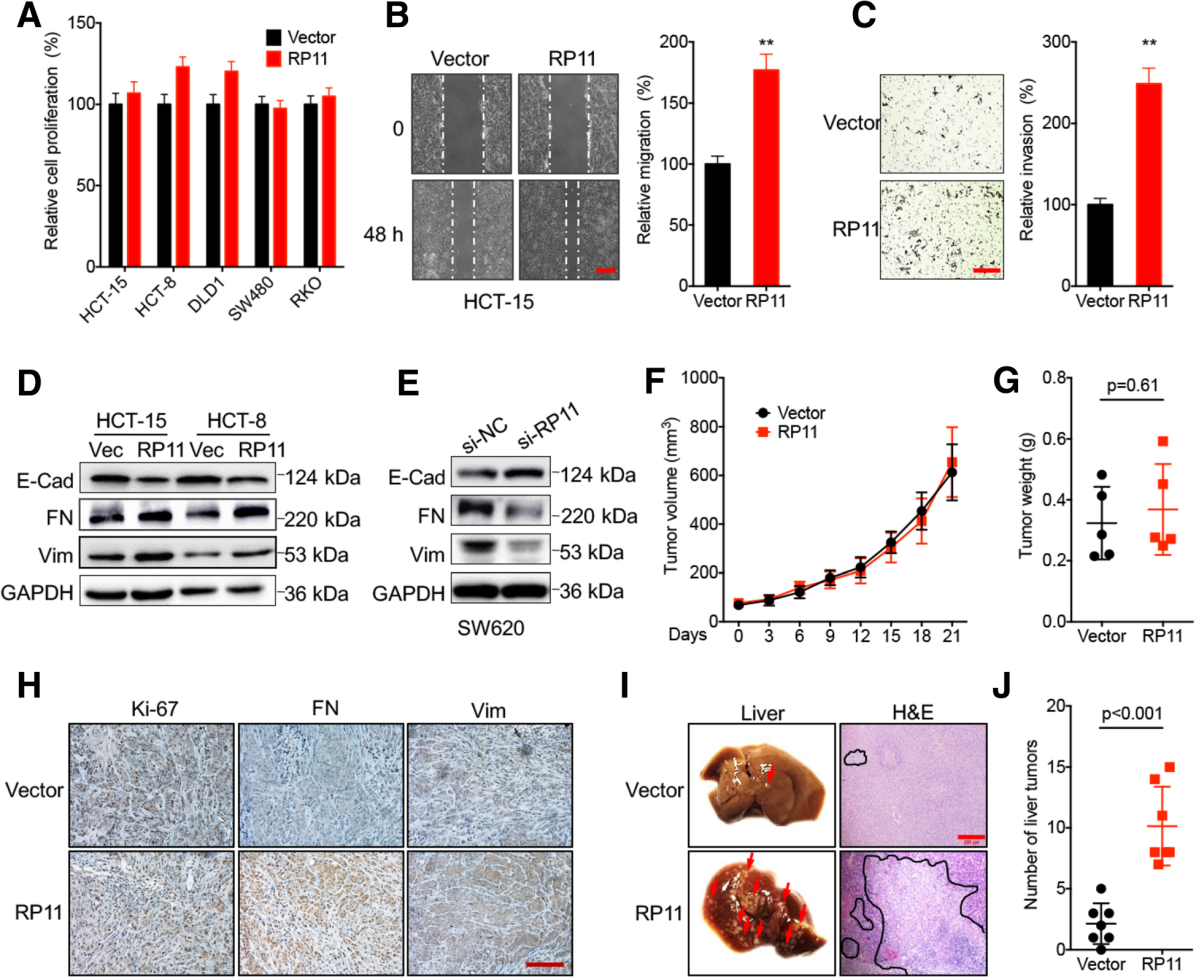
RP11 triggers the dissemination of CRC cells both in vitro and in vivo. **a** CRC cells were transfected with the vector control or pcDNA/RP11 for 48 h, and proliferation was measured with a CCK-8 kit. **b** The wound healing of HCT-15 RP11 stable overexpression and control cells was recorded (*left*) and quantitatively analysed (*right*). **c** The in vitro invasion of HCT-15 RP11 stable overexpression and control cells was recorded (*left*) and quantitatively analysed (*right*). **d** The expression of EMT-related markers of HCT-15 or HCT-8 RP11 stable overexpression and control cells was verified by western blot analysis. **e** After transfection with si-NC or si-RP11 for 48 h, the expression of EMT markers in SW620 cells was verified by western blot analysis. **f** Tumour growth curves of HCT-15 RP11 stable overexpression and control cells in xenograft models at the indicated time intervals. **g** Weights of tumours derived from HCT-15 RP11 stable overexpression or control cells in xenograft models at the end of the experiment. **h** IHC analysis of Ki67-, vimentin- or fibronectin-stained paraffin-embedded sections obtained from xenografts. (I).HCT-15 RP11 stable overexpression and control cells were injected into nude mice via the tail vein. Representative images and H&E staining of metastatic liver tumours are shown. **j** The number of metastatic sites of tumours derived from HCT-15 RP11 stable overexpression or control cells was quantitatively analysed. Data are presented as the mean ± SD from three independent experiments. Bar = 200 μm. *p < 0.05, **p < 0.01 compared with control

The potential biological roles of RP11 in CRC progression were investigated. We overexpressed RP11 in HCT-15, HCT-8, DLD1, SW480 and RKO cells (RP11 low expression cells, Additional file 1: Figure S2 A). CCK-8 analysis showed RP11 overexpression had no significant effect on the proliferation of these cells (Fig. 2 a). Consistently, RP11 silencing in SW620 or HCT-116 cells (RP11 high expression cells, Additional file 1: Figure S2 B) also had no significant effect on cell proliferation (Additional file 1: Figure S2 C). The colony formation analysis showed that RP11 overexpression had no significant effect on colony formation of HCT-15 or HCT-8 cells (Additional file 1: Figure S2 D). Flow cytometry showed that RP11 overexpression had no significant effect on HCT-15 or HCT-8 cell cycle progression (Additional file 1: Figure S2 E). In addition, RP11 overexpression had no significant effect on stress-induced apoptosis, doxorubicin sensitivity, rhodamine123 efflux or ROS generation in HCT-15 (Additional file 1: Figure S2 F~I) or HCT-8 (data not shown) cells.

The effects of RP11 on the in vitro migration and invasion of CRC cells were evaluated. A wound healing analysis revealed that RP11 overexpression triggered the migration of both HCT-15 (Fig. 2 b) and HCT-8 (Additional file 1: Figure S2 J) cells. Transwell analysis confirmed that RP11 can increase the in vitro invasion of HCT-15 cells (Fig. 2 c). RP11 silencing inhibited the in vitro migration (Additional file 1: Figure S2 K) and invasion (Additional file 1: Figure S2 L) of SW620 cells. CRC cells overexpressing RP11 assumed their spindle-like fibroblast appearance and lost their cobblestone-like epithelial morphology (Additional file 1: Figure S2 M), suggesting that RP11 may regulate EMT and cancer metastasis. This was confirmed by western blot analysis, which showed a decrease in the expression of epithelial cell marker E-Cadherin (E-Cad) and an increase in the expression of mesenchymal cell markers fibronectin (FN) and Vimentin (Vim) in HCT-15 and HCT-8 cells transfected with RP11 (Fig. 2 d). RP11 silencing impaired EMT progression in SW620 (Fig. 2 e) and HCT-116 (Additional file 1: Figure S2 N) cells. Collectively, our data suggested that RP11 can induce the migration, invasion and EMT of CRC cells.

To evaluate the in vivo effects of RP11 on tumour development, we examined the expression levels of EMT-related markers in RP11-overexpressing HCT-15 tumour xenografts in nude mice. At the end of the experiment, the tumour sizes, volumes and weights in the RP11 group were comparable to those in the control group (Fig. 2 f, g). This was confirmed by IHC analysis of the expression of Ki67, a nuclear antigen expressed in proliferating cells, and the Ki67 level was comparable between the RP11 and control groups (Fig. 2 h). The IHC data showed that RP11 increased the levels of Vim and FN in HCT-15 tumour xenografts (Fig. 2 h).

To further determine the impacts of RP11 on in vivo metastasis, equal numbers of HCT-15 RP11 stable overexpression and control cells (1 × l0^6^ in 100 μl) were injected into BALB/c nude mice via the tail vein, and distant liver metastasis was analysed. Eight weeks after injection, the experiment was terminated, and the liver was analysed for the presence of metastatic tumours. As shown in Fig. 2 i & j, the numbers and sizes of the liver tumours derived from RP11-overexpressing HCT-15 cells were significantly greater than those derived from the control cells. Collectively, our data showed that RP11 can enhance the in vitro and in vivo dissemination of CRC cells and induce EMT.

### Upregulation of Zeb1 mediates the RP11-induced dissemination of CRC cells

LncRNA can activate the transcription of closely located genes *in cis* by promoting chromatin looping from transcriptional enhancers [25, 26]. We therefore investigated the effects of RP11 on its nearby transcripts, including NUDT12, C5orf30, PPIP5K2, GIN1, RP11-6 N13.1, and CTD-2374C24 (Additional file 1: Figure S1 B). The expression levels of the detected genes showed no significant difference between the HCT-15 RP11 stable and control cells (Additional file 1: Figure S3 A). In SW620 cells, RP11 knockdown also had no effect on the expression of its nearby transcripts (Additional file 1: Figure S3 B). Thus, the biological functions of RP11 may not be related to the *cis* regulatory function.

**Fig. 3:**
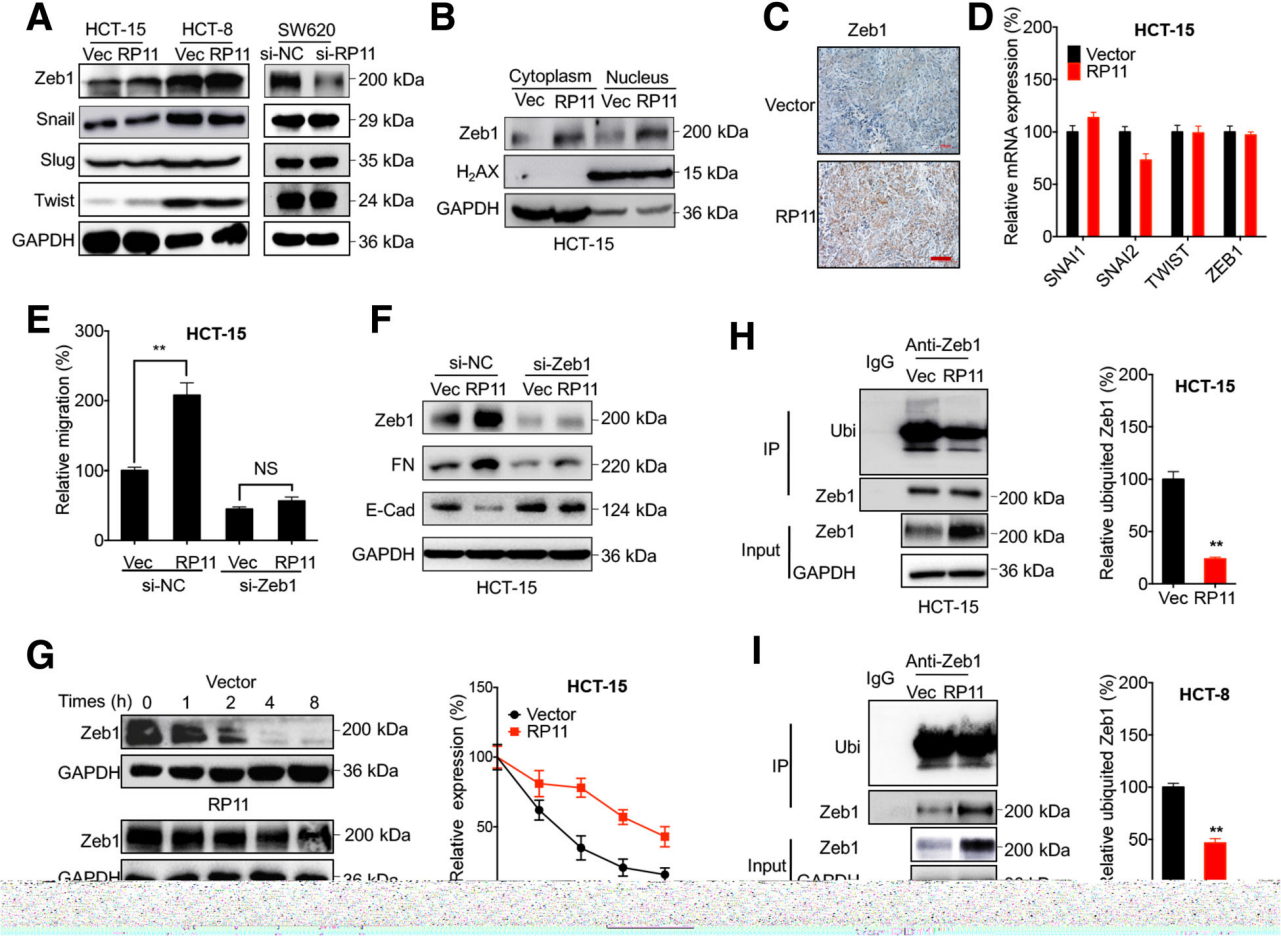
Upregulation of Zeb1 mediates the RP11-induced dissemination of CRC cells. **a**. The expression levels of EMT-TFs in HCT-15 or HCT-8 RP11 stable overexpression and control cells were verified by western blot analysis. After transfection with si-NC or si-RP11 for 48 h, the expression levels of EMT-TFs in SW620 cells were verified by western blot analysis. **b** Zeb1 expression in subcellular fractions of HCT-15 RP11 stable overexpression and control cells was verified by western blot analysis. **c** IHC analysis of Zeb1-stained paraffin-embedded sections obtained from xenografts. **d** The mRNA expression levels of EMT-TFs in HCT-15 RP11 stable overexpression and control cells were verified by qRT-PCR. **e** After transfection with si-NC or si-Zeb1 for 48 h, the wound healing of HCT-15 RP11 stable overexpression and control cells was quantitatively analysed. **f** After transfection with si-NC or si-Zeb1 for 48 h, the EMT markers of HCT-15 RP11 stable overexpression and control cells were detected by western blot analysis. **g** After treatment with 100 μg/ml CHX for the indicated times, Zeb1 expression in HCT-15 RP11 stable overexpression and control cells was detected by western blot analysis (*left*) and quantitatively analysed (*right*). **h** & **i** Zeb1 in HCT-15 or HCT-8 RP11 stable overexpression and control cells was immunoprecipitated for the detection of ubiquitylation. Data are presented as the mean ± SD from three independent experiments. Bar = 200 μm. **p < 0.01 compared with control

EMT-TFs such as Snail, Slug, Twist and Zeb1 can regulate the progression of EMT by targeting E-Cad expression [27]. To investigate the mechanisms responsible for the RP11-induced dissemination of CRC cells, we analysed the effects of RP11 on the expression of EMT-TFs in CRC cells. The results showed that RP11 overexpression increased the expression of Zeb1 in both HCT-15 and HCT-8 cells, while si-RP11 downregulated the expression of Zeb1 in SW620 and HCT-116 cells (Fig. 3 a and Additional file 1: Figure S3 C). RP11 overexpression or knockdown had no effect on the expression of Snail, Slug or Twist (Fig. 3 a and Additional file 1: Figure S3 C). The subcellular fraction showed that RP11 overexpression increased the nuclear accumulation of Zeb1 in HCT-15 cells (Fig. 3 b). Consistently, RP11 increased Zeb1 expression in HCT-15 tumour xenografts (Fig. 3 c). Intriguingly, neither RP11 overexpression in HCT-15 (Fig. 3 d) nor knockdown in SW620 (Additional file 1: Figure S3 D) cells had significant effect on the mRNA levels of tested EMT-TFs. Consistently, RP11 overexpression had no effect on the mRNA expression of Zeb1 in Caco2, HT-29, SW480, DLD1, or RKO cells (Additional file 1: Figure S3 E).

Although Zeb1 has been well demonstrated to induce the EMT of cancer cells, including CRC cells, by inhibiting E-Cad [17, 28], the role of Zeb1 in the RP11-induced dissemination of CRC cells was unknown and thus investigated. A wound healing analysis showed that Zeb1 knockdown attenuated RP11-induced cell migration (Fig. 3 e, Additional file 1: Figure S3 F). Western blot analysis confirmed that Zeb1 knockdown attenuated RP11-induced upregulation of FN and downregulation of E-Cad (Fig. 3 f).

The results indicated that RP11 may increase Zeb1 expression via post-translational regulation. This was confirmed by data showing that the half-life of the Zeb1 protein in HCT-15 (Fig. 3 g) and HCT-8 (Additional file 1: Figure S3 G) RP11 stable overexpression cells was significantly greater than that in their corresponding control cells. Because ubiquitylation of Zeb1 is critical for its stabilization [29], we hypothesized that RP11 modified the ubiquitylation level of Zeb1. Immunoprecipitation results showed that RP11 can significantly decrease the ubiquitylation of Zeb1 in both HCT-15 (Fig. 3 h) and HCT-8 (Fig. 3 i) cells. Collectively, our present data suggested that the post-translational upregulation of Zeb1 is involved in the RP11-induced dissemination of CRC cells.

### Downregulation of Siah1 and Fbxo45 mediates RP11-induced upregulation of Zeb1

Because lncRNAs can directly intact with proteins and thereby regulate protein stability [25, 30], the binding of Zeb1 to RP11 was investigated by RIP-PCR. The data showed that immunoprecipitation (IP) of Zeb1 had no significant effect on RP11 recruitment in either HCT-15 or HCT-8 cells (Additional file 1: Figure S4 A). In addition, Zeb1 overexpression had no effect on the RP11 expression in either HCT-15 or HCT-8 cells (Additional file 1: Figure S4 B). Consistently, the RP11 pull-down/MS analysis did not show binding between RP11 and Zeb1 in either the HCT-15 control or RP11 stable overexpression cells (Table S4). This suggested that the RP11-induced upregulation of Zeb1 is not due to a direct interaction. GSK-3β, β-catenin, p65, MAPK/ERK, p38-MAPK, PI3K/Akt, and STAT3 have been reported to regulate Zeb1 expression and EMT [31]. However, no significant variation was observed in the total and phosphorylated levels of these signalling molecules between HCT-15 RP11 stable overexpression and control cells (Additional file 1: Figure S4 C).

**Fig. 4:**
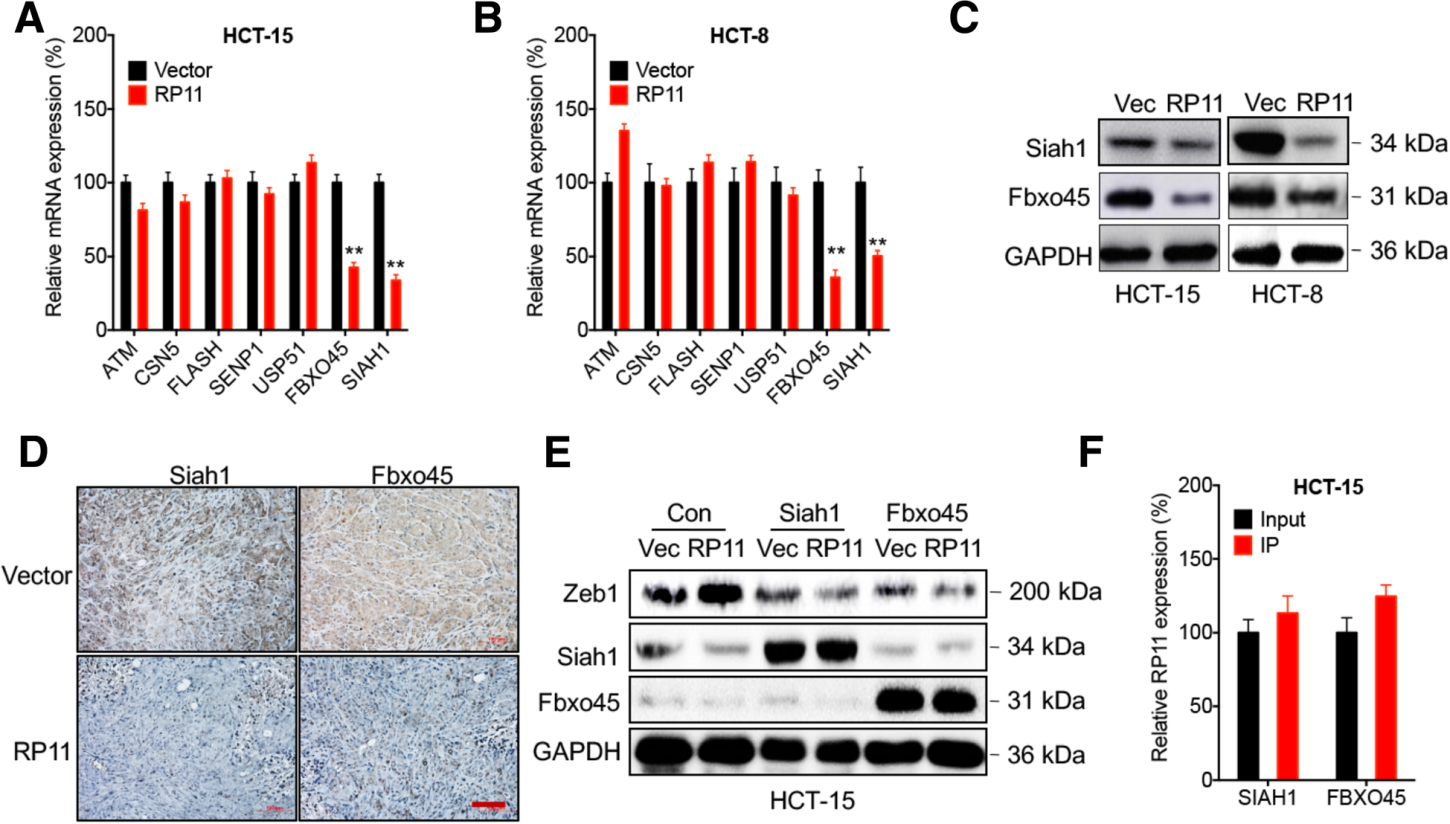
Downregulation of Siah1 and Fbxo45 mediates the RP11-induced upregulation of Zeb1. **a** & **b** The mRNA expression levels of 7 reported target proteins related to Zeb1 stability in HCT-15 **a** or HCT-8 (**b**) RP11 stable overexpression and control cells were determined by qRT-PCR. **c** The protein expression of Siah1 and Fbxo45 in HCT-15 or HCT-8 RP11 stable overexpression and control cells was determined by western blot analysis. **d** IHC analysis of Siah1- or Fbxo45-stained paraffin-embedded sections obtained from HCT-15 RP11 stable overexpression and control xenografts. **e** HCT-15 cells were transfected with vector control, pcDNA/RP11, pcDNA/Siah1, or Fbxo45 alone or together for 48 h, protein expression was verified by western blot analysis. **f** RIP-PCR was performed to analyse the relative enrichment of RP11 by use of an antibody against Siah1 or Fbxo45 in HCT-15 cells. Data are presented as the means ± SD from three independent experiments. Bar = 200 μm. **p < 0.01 compared with control

To systematically investigate the specific factors involved in the RP11-induced stabilization of Zeb1 in CRC cells, we examined the mRNA expression levels of 7 reported factors in the ubiquitin–proteasome system, which can post-translationally regulate the stability of Zeb1 (summarized in Table S5). The results indicated that RP11 overexpression significantly (*p* < 0.05) decreased the expression levels of Siah1 and Fbxo45 but had no significant effect on other factors in either HCT-15 (Fig. 4 a) or HCT-8 (Fig. 4 b) cells. This was confirmed by a western blot analysis showing that RP11 overexpression downregulated the expression of Siah1 and Fbxo45 in both HCT-15 and HCT-8 cells (Fig. 4 c). Consistently, RP11 decreased the expression of Siah1 and Fbxo45 in HCT-15 tumour xenografts (Fig. 4 d).

To verify the roles of Siah1 and Fbox45 in the expression of Zeb1, we overexpressed Siah1 and Fbxo45 in HCT-15 cells (Fig. 4 e). The results showed that overexpression of Siah1 and Fbxo45 attenuated the RP11-induced upregulation of Zeb1 in HCT-15 cells (Fig. 4 e). However, RIP-PCR showed that RP11 had no significant effect on the recruitment of Siah1 or Fbxo45 protein in HCT-15 cells (Fig. 4 f). Consistently, the RP11 pull-down/MS analysis did not show binding between RP11 and Siah1 or Fbxo45 in HCT-15 cells (Table S4). This result suggested that RP11 downregulates the mRNA levels of Siah1 and Fbxo45 but does not bind to the Siah1 or Fbxo45 protein.

### RP11 regulates Siah1 and Fbxo45 expression by forming the RP11-hnRNPA2B1-mRNA complex

**Fig. 5:**
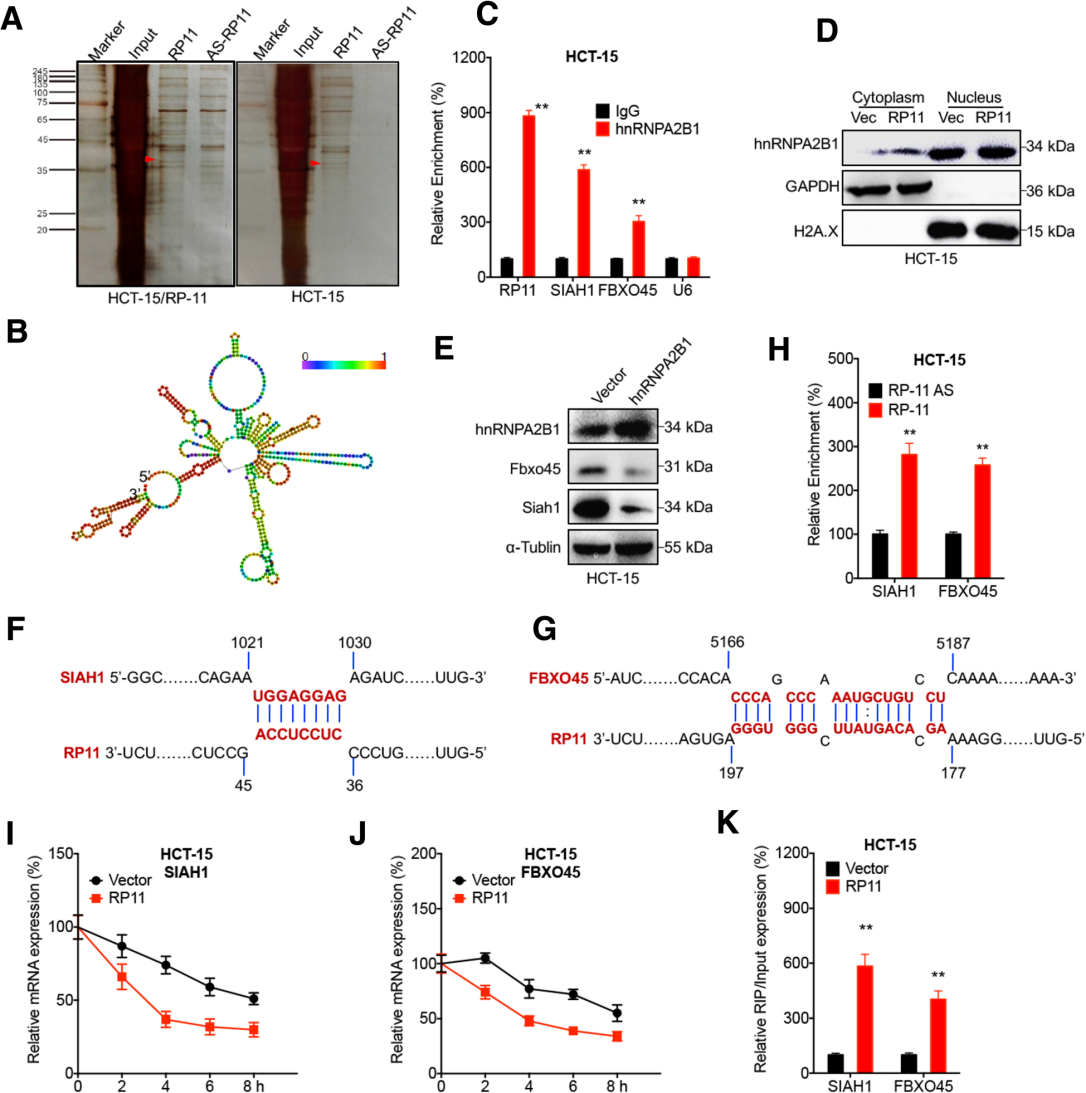
RP11 regulates Siah1 and Fbxo45 expression by forming the RP11-hnRNPA2B1-mRNA complex. **a** RNA pull-down analysis and MS identified hnRNPA2B1 as the specific protein interacting with RP11 in both HCT-15 and HCT-15 RP11 stable overexpression cells. The red arrow shows the position of hnRNPA2B1. **b** The secondary structure of RP11 was predicted (http://rna.tbi.univie.ac.at/). The red colour indicates strong confidence for the prediction of each base. **c** RNA pull-down detection of the interaction between hnRNPA2B1 and RP11, Siah1, or Fbxo45 in HCT-15 cells. **d** hnRNPA2B1 expression in the cytoplasmic and nuclear fractions of HCT-15 RP11 stable overexpression and control cells were analysed by western blot. **e** HCT-15 cells were transfected with pcDNA (vector) or pcDNA/hnRNPA2B1 for 24 h, and the expression of Siah1 and Fbxo45 was verified by western blot analysis. **f** &**g** The computational prediction of the interaction between RP11 and the Siah1 (**f**) or Fbxo45 (**g**) mRNA based on IntaRNA 2.0 (http://rna.informatik.uni-freiburg.de/IntaRNA/Input.jsp) [53]. **h** After in vitro transcription to generate biotin-labelled RP-11 and RP-11 AS, RIP-PCR was performed to analyse the relative enrichment of Siah1 or Fbxo45 mRNA on RP11 in HCT-15 cells. **i** & **j** After treatment with Act-D for the indicated times, the mRNA levels of Siah1 (**i**) or Fbxo45 (**j**) in HCT15 RP11 stable overexpression and control cells were measured by qRT-PCR. **k** Binding between hnRNPA2B1 and Siah1 mRNA or between hnRNPA2B1 and Fbxo45 mRNA in HCT-15 RP11 stable overexpression and control cells was analysed by RIP-PCR. Data are presented as the mean ± SD from three independent experiments. **p < 0.01 compared with control

To investigate the potential mechanisms of the RP11-regulated mRNA expression of Siah1 and Fbxo45, we performed RNA pull-down assays followed by MS with biotinylated RP11 and antisense RP11 as a negative control. Among the identified proteins summarized in Table S4, hnRNPA2B1 was identified as a protein that directly interacted with RP11 (Fig. 5 a) and has been reported to shorten mRNA half-lives [32]. RIP analysis verified the interaction between hnRNPA2B1 and RP11 in HCT-15 cells (Fig. 5 b&c)*.*

hnRNPA2B1 is an RNA binding protein (RBP) and localizes in both the cytoplasm and nucleus. Our data showed that RP11 overexpression increased the cellular localization of hnRNPA2B1 in the cytoplasm in both HCT-15 (Fig. 5 d) and HCT-8 (Additional file 1: Figure S5 A) cells. RIP-PCR showed that hnRNPA2B1 could recruit both Siah1 and Fbxo45 mRNA in HCT-15 cells (Fig. 5 c). Furthermore, hnRNPA2B1 overexpression decreased the mRNA (Additional file 1: Figure S5 B) and protein (Fig. 5 e) expression of Siah1 and Fbxo45 in HCT-15 cells.

Computational analysis revealed that RP11 could directly bind to the CDS of Siah1 (Fig. 5 f) and the 3’UTR of Fbxo45 (Fig. 5 g). In vitro transcription and RIP-PCR confirmed that RP11 could directly bind to the mRNA of Siah1 and Fbxo45 in HCT-15 cells (Fig. 5 h). RP11 overexpression significantly downregulated the mRNA stability of Siah1 (Fig. 5 i) and Fbxo45 (Fig. 5 j) in HCT-15 cells.

We further investigated whether binding between hnRNPA2B1 and the mRNA of Siah1 and Fbxo45 was RP11 dependent. RIP-PCR showed that the binding between hnRNPA2B1 and the mRNA of Siah1 and Fbxo45 in the HCT-15 RP11 stable overexpression cells was significantly greater than that in the control cells (Fig. 5 k). Consistently, RP11 knockdown decreased the binding between hnRNPA2B1 and the mRNA of Siah1 and between hnRNPA2B1 and the mRNA of Fbxo45 in HCT-15 cells (Additional file 1: Figure S5 C). These data suggested that RP11 regulates Siah1 and Fbxo45 expression by forming the RP11-hnRNPA2B1-mRNA complex.

### m^6^A modification is involved in the upregulation of RP11 in CRC cells

The epigenetic mechanisms responsible for the upregulation of RP11 in CRC cells were investigated. First, treatment with 5-aza-dC (a DNA methyltransferase inhibitor) had no significant effect on RP11expression in either HCT-15 or HCT-8 cells (Additional file 1: Figure S6 A), suggesting that DNA methylation might not be involved in RP11 expression in CRC cells. The role of histone acetylation in RP11 expression was investigated by treating HCT-15 cells with specific inhibitors of HDAC1, 3, 4, 6 and 8 or broad-spectrum HDAC inhibitors such as SAHA and NaB. The data showed that these HDAC inhibitors had no significant effect on RP11 expression in HCT-15 cells (Additional file 1: Figure S6 B). This was confirmed by data showing that overexpression of HDAC6 and HDAC8 had no effect on RP11 expression in HCT-15 cells (Additional file 1: Figure S6 C).

**Fig. 6:**
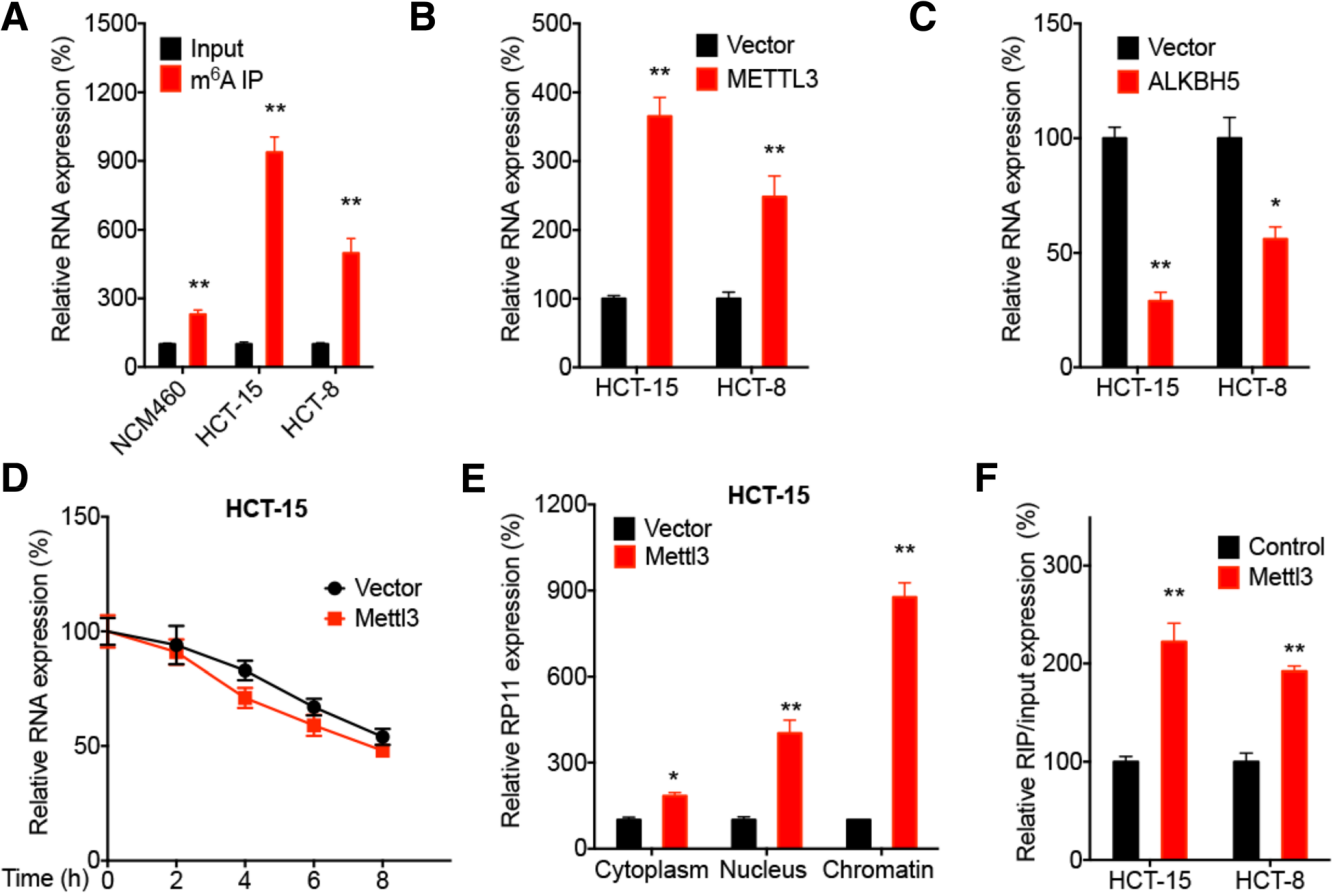
The m^6^A modification is involved in the upregulation of RP11 in CRC cells. **a** m^6^A RIP-qPCR analysis of RP11 in HCT-15, HCT-8 and NCM460 cells. **b** After transfection with vector control or ppB/Mettl3 for 24 h, RP11 expression was measured by qRT-PCR. **c** After transfection with vector control or pcDNA/Alkbh5 for 24 h, RP11 expression was measured by qRT-PCR. **d** After transfection with vector control or ppB/Mettl3 for 24 h, HCT-15 cells were further treated with Act-D for the indicated times, and RP11 expression was measured by qRT-PCR. **e** After transfection with vector control or ppB/Mettl3 for 24 h, the cytoplasmic, nuclear, and chromatin fractions of HCT-15 cells were separated for RNA extraction and qRT-PCR. **f** After transfection with vector control or ppB/Mettl3 for 24 h, binding between RP11 and hnRNPA2B1 in HCT-15 and HCT-8 cells was analysed by RIP-PCR using an antibody against hnRNPA2B1. Data are presented as the mean ± SD from three independent experiments. *p < 0.05, **p < 0.01 compared with control

The *N6*-methyladenosine (m^6^A) modification modulates all stages of the RNA life cycle, such as RNA processing, nuclear export and translation [33, 34], and thereby regulates the expression and functions of RNAs, including lncRNAs. m^6^A RNA-immunoprecipitation (RIP) qPCR showed 9.3- and 5.0-fold enrichment in m^6^A antibody levels of RP11 in HCT-15 and HCT-8 cells, respectively (Fig. 6 a), while the level of enrichment (2.3-fold) in NCM460 cells was significantly less than that in CRC cells (Fig. 6 a). We found that overexpression of Mettl3 (Additional file 1: Figure S6 D), the key m^6^A methyltransferase (“writer”) in mammalian cells [35, 36], increased RP11 expression in both HCT-15 and HCT-8 cells (Fig. 6 b). Consistently, overexpression of ALKBH5 (Additional file 1: Figure S6 E), the demethylase of m^6^A, decreased RP11 expression (Fig. 6 c). These data indicated that m^6^A positively regulates RP11 expression in CRC cells.

We then evaluated the possible mechanisms involved in the m^6^A-regulated expression of RP11 in CRC cells. By treating cells with Act-D to terminate transcription, our data revealed that Mettl3 overexpression had no significant effect on the half-life of RP11 in HCT-15 cells (Fig. 6 d). The results of subcellular fractionation analysis showed that Mettl3 overexpression could markedly increase the localization of RP11 to chromatin (Fig. 6 e), which might be because Mettl3 can increase the stability of nascent RP11. However, Mettl3 overexpression had no effect on the mRNA expression of Siah1 or Fbxo45 in HCT-15 cells (Additional file 1: Figure S6 F). Furthermore, Mettl3 overexpression increased binding between RP11 and hnRNPA2B1 in both HCT-15 and HCT-8 cells (Fig. 6 f), which might be due to Mettl3 increasing RP11 expression and hnRNPA2B1 is a m^6^A reader for the RNA processing events [37]. These data suggested that the m^6^A modification can increase RP11 expression in CRC cells by increasing RP11 nuclear accumulation.

### The m^6^A/RP11/Zeb1 axis and in vivo progression of CRC

**Fig. 7:**
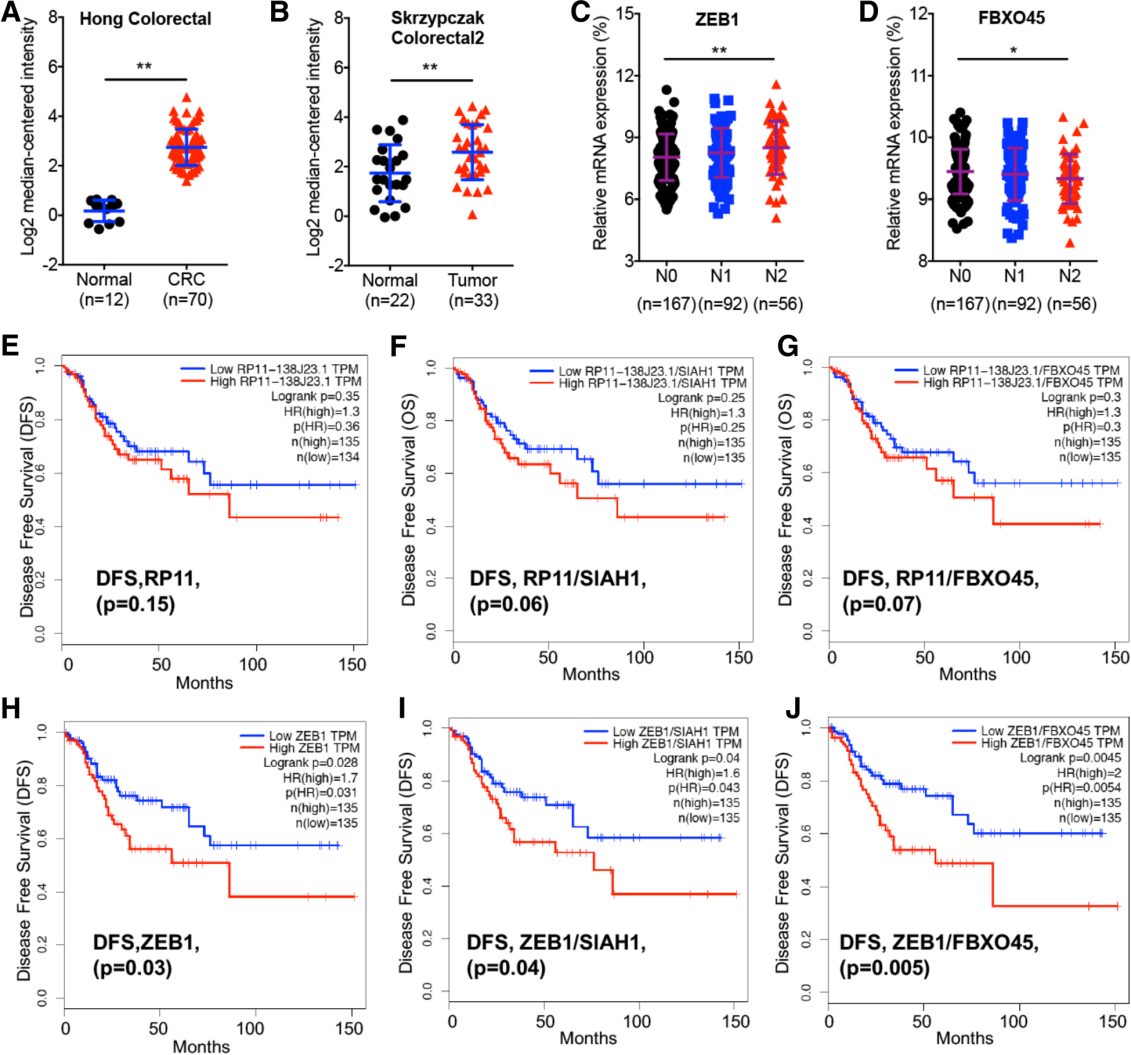
The m^6^A/RP11/Zeb1 axis and in vivo progression of CRC. **a** & **b** The relative mRNA expression of Zeb1 in two Oncomine datasets: Hong Colorectal (**a**), and Skrzypczak Colorectal 2 (**b**). **c** & **d** The relative mRNA expression of Zeb1 (**c**) and Fbxo45 (**d**) in patients with stage N0, N1, and N2 CRC based on data available from TCGA database. **e** DFS of CRC patients with high (*n* = 135) and low (*n* = 134) levels of RP11 was plotted according to the Kaplan-Meier method. **f** DFS of CRC patients with high (*n* = 135) and low (*n* = 135) levels of RP11/Siah1 was plotted according to the Kaplan-Meier method. **g** DFS of CRC patients with high (*n* = 135) and low (*n* = 135) levels of RP11/Fbxo45 was plotted according to the Kaplan-Meier method. (H) DFS of CRC patients with high (*n* = 135) and low (*n* = 134) levels of Zeb1 was plotted according to the Kaplan-Meier method. (I) DFS of CRC patients with high (*n* = 135) and low (*n* = 135) levels of Zeb1/Siah1 was plotted according to the Kaplan-Meier method. **j** DFS of CRC patients with high (*n* = 135) and low (*n* = 135) levels of Zeb1/Fbxo45 was plotted according to the Kaplan-Meier method. *p < 0.05, **p < 0.01 compared with control

At this point, we asked whether there was a link between m^6^A methylation-regulated RP11, its downstream molecules Siah1, Fbxo45, and Zeb1, and clinical CRC development. Zeb1 expression in CRC tissues was significantly (*p* < 0.01) greater than that in normal tissues, according to Hong Colorectal (Fig. 7 a) and Skrzypczak Colorectal 2 data (Fig. 7 b) from the Oncomine database. Significantly increased Zeb1 was observed in patients with N2 stage CRC compared to patients with N0 stage CRC (Fig. 7 c). Consistently, decreased expression of Fbxo45 was observed in patients with N2 stage CRC compared to patients with N0 stage CRC (Fig. 7 d). In addition, Mettl3 expression in patients with N2 stage CRC was significantly greater than that in patients with N1 stage CRC (Additional file 1: Figure S7 A). However, there was no significant difference for Siah1 between patients with N0, N1 or N2 stage CRC (Additional file 1: Figure S7 B). This finding suggested an increasing trend for METTL3 and Zeb1 expression and a decreasing trend for Fbxo45 expression during the malignant transformation of CRC. We further verified the co-expression relationship for RP11-regulated CRC progression. We found that RP11 expression was significantly negatively correlated with ALKBH5 expression in CRC patients (Additional file 1: Figure S7 C). This confirmed that the m^6^A can regulate the expression of RP11, further, RP11 regulated Siah1-Fbxo45/Zeb1 was involved in the development of CRC.

Using the online Kaplan-Meier plotter bioinformatics tool, we found that colon cancer patients with increased RP11 expression showed reduced disease-free survival (DFS, Fig. 7 e) and overall survival (OS, Additional file 1: Figure S7 D), with no significant difference (*p* > 0.05). When RP11 expression was normalized to that of Siah1 (the relative levels of RP11 to that of Siah1) or Fbxo45, there was a trend towards significance for the reduced DFS of colon patients with higher RP11/Siah1 (Fig. 7 f) or RP11/Fbxo45 levels (Fig. 7 g) compared with patients with lower values. Similarly, there was a trend towards significance for the reduced OS of colon cancer patients with higher RP11/Siah1 (Additional file 1: Figure S7 E) or RP11/Fbxo45 levels (Additional file 1: Figure S7 F) compared with those with lower values.

We found that colon cancer patients with increased Zeb1 expression showed reduced DFS (Fig. 7 h) with significant difference (*p* < 0.05). When Zeb1 expression was normalized to that of Fbxo45 (Fig. 7 j), but not Siah1 (Fig. 7 i), the DFS of colon cancer patients with higher Zeb1/Siah1 levels (Fig. 7 j) was statistically significantly reduced compared to patients with lower values. Similarly, colon cancer patients with increased Zeb1 expression showed significantly (*p* < 0.05) reduced OS compared with patients with the low levels (Additional file 1: Figure S7 G). Normalization to Fbxo45 (Additional file 1: Figure S7 I), but not Siah1 (Additional file 1: Figure S7 J), further significantly reduced the OS of colon cancer patients. These results suggest that the m^6^A/RP11/Zeb1 axis triggers the in vivo progression of CRC.

## Discussion

The application of next-generation sequencing has revealed that thousands of lncRNAs are involved in the progression of human disease. Several lncRNAs have been reported to play key roles in cancer developmental processes, including proliferation, survival, migration or genomic stability [25]. Among the few lncRNAs that have been functionally characterized, several have been linked to cancer cell invasion and metastases [38, 39]. Regarding CRC progression, lncRNAs have been reported to regulate cell survival [40], tumorigenicity [10], and asymmetric stem cell division [41]. By using microarray analysis and functional screening, we show that lncRNA RP11, which is upregulated by m^6^A methylation, can trigger the migration, invasion and EMT of CRC cells via post-translational upregulation of the EMT-TF Zeb1.

Our study highlights the function and mechanisms of RP11 in regulating CRC metastasis. Among the 8 simultaneously upregulated lncRNAs between stage I and normal tissues, stage IV and normal tissues, and stage IV and stage I tissues, RP11 expression in CRC tissues was not only greater than that in adjacent normal tissues but also higher than that in other cancers, suggesting that RP11 might be a specific target for CRC diagnosis and therapy. By screening for its potential roles in cell proliferation, colony formation, cell cycle progression, apoptosis, drug sensitivity/accumulation, and ROS generation via gain- and loss-of-function assessments, we found that RP11 can trigger the migration, invasion and EMT of CRC cells both in vitro and in vivo. This was evidenced by the observed upregulation of FN and vim and downregulation of E-Cad. Together with published reports of cancer metastasis-related lncRNAs, such as lncRNA-ATB [38], SChLAP1 [39], NKILA [30], and PNUTS [42], our study confirms the regulatory roles of lncRNAs in EMT and cancer metastasis. High RP11 expression correlates with positive lymph node metastasis and advanced TNM stage, suggesting that RP11 can be a strong predictor of CRC metastasis and prognosis.

We find that the post-translational regulation of Zeb1 plays an essential role in the RP11-triggered dissemination of CRC. Zeb1 is a well-known and powerful EMT-TF that promotes EMT, metastasis, and the generation of cancer stem cells in many types of malignancies, including CRC [28, 43]. We findd that RP11 has no effect on mRNA expression but increases the protein expression of Zeb1 in CRC cells by increasing Zeb1 protein stability and decreasing Zeb1 ubiquitination. By screening for factors responsible for the stability of Zeb1 in cancer cells, we confirm that the downregulation of Siah1 and Fbxo45 mediates the RP11-induced stabilization of Zeb1 in CRC cells. As ubiquitin E3 ligases, Siah1 and Fbxo45 can induce Zeb1 degradation through the ubiquitin-proteasome pathway [44, 45].

lncRNAs can modulate the stability and nuclear turnover of specific mRNAs via RBPs and miRNAs [5]. In this work, the RP11-hnRNPA2B1-mRNA complex downregulates the mRNA stability of Siah1 and Fbxo45 in CRC cells. RP11 can be detected in both the cytoplasm and nucleus in CRC cells. The actions of RP11 towards decreasing mRNA stability through hnRNPA2B1 can be attributed to the cytoplasmic localization RP11. This is supported by the observation that RP11 increases the cytoplasmic accumulation of hnRNPA2B1, while hnRNPA2B1 overexpression decreases the expression of Siah1 and Fbxo45. Several existing studies have demonstrated that lncRNAs form complexes with RBPs and then trigger mRNA decay [32, 46]. HnRNPA2B1 is known to form complexes with lncRNAs and is emerging as an important mediator of lncRNA-induced transcriptional repression [47]. Recently, lncRNA *lncHC*-binding hnRNPA2B1 has been reported to directly bind to the *Cyp7a1* and *Abca1* mRNAs and reduce their expression levels in hepatocytes [32]. In addition, hnRNPA2B1 interaction with lncRNA RMST may indicate the participation of the lncRNA in alternative splicing, mRNA trafficking, and neuronal cell survival [48]. Although our findings link RP11 and hnRNPA2B1 to suppression of mRNA stability, the detailed molecular mechanism is not currently understood in depth. This might be because hnRNPA2B1 can recruit factors involved in the mRNA degradation pathway (such as P bodies) to accelerate mRNA degradation.

Finally, we explore whether m^6^A methylation, but not DNA methylation or histone acetylation, is involved in the upregulation of RP11 in CRC cells. m^6^A methylation involvement is evidenced by the observation that RP11 is significantly enriched with m^6^A-RIP and that Mettl3 significantly increases RP11 expression in CRC cells. As one of the most common RNA modifications, m^6^A can be found on almost all types of RNAs; can modulate all stages of the RNA life cycle, such as RNA processing, nuclear export and translation [33, 34]; and can therefore regulate cancer progression processes, such as cell proliferation [49] and tumorigenesis [50]. However, investigations of the functions of m^6^A in lncRNAs are few. One recent study first revealed an m^6^A-dependent model of the lincRNA/miRNA interaction in which the m^6^A modification of *linc1281* was required for the direct binding of let-7 to *linc1281* in embryonic stem cells (ESCs) [51]. We reveal that m^6^A could increase RP11 accumulation in the nucleus and on chromatin. We find that Mettl3 overexpression could increase binding between hnRNPA2B1 and RP11 in CRC cells, which might be due to m^6^A-induced alterations in the local RNA structure and enhancements in the RNA binding of hnRNPs [52]. Considering that knowledge of the mechanism of RNA methylation is still in its infancy, additional discoveries of regulatory patterns mediated by m^6^A on the biogenesis and functions of lncRNA are worth verifying in the future.

In conclusion, our findings demonstrate the pro-metastatic role of lncRNA RP11 in the dissemination of CRC cells. We have discovered that RP11 post-translationally stimulates Zeb1 expression via downregulation of the mRNA expression of Siah1 and Fbxo45 by binding to hnRNPA2B1. Furthermore, m^6^A modification may increase RP11 expression and function in CRC cells and tissues. Considering the high and specific levels of RP11 in CRC tissues, our present study provides a potent target that may serve as a predictive marker of metastasis and as an effective target for anti-metastatic therapies for CRC patients.

## Additional file

Additional file 1: **Figure S1.** RP11 is increased during the tumourigenesis and progression of CRC. **Figure S2.** RP11 triggers the dissemination of CRC cells both in vitro and in vivo. **Figure S3.** Upregulation of Zeb1 mediates RP11-triggered dissemination of CRC cells. **Figure S4.** Downregulation of Siah1 and Fbxo45 mediates RP11-induced upregulation of Zeb1. **Figure S5.** RP11 regulates Siah1 and Fbxo45 expression by forming the RP11-hnRNPA2B1-mRNA complex. **Figure S6.** The m6A modification is involved in the upregulation of RP11 in CRC cells. **Figure S7.** The m6A/RP11/Zeb1 axis and in vivo progression of CRC. (DOCX 14708 kb)

Additional file 2: **Table S1.** The clinic pathological features of clinical CRC tissues (*n* = 32). **Table S2.** Sequences of primers. **Table S3.** The information of 8 lncRNAs. **Table S4.** The protein information of RP11 pull down/MS analysis. **Table S5.** Factors related to the stability of Zeb1 in cancer cells. (ZIP 279 kb)

## Abbreviations

Act-D: Actinomycin D
CHX: Cycloheximide
CRC: Colorectal cancer
DFS: Disease-free survival
E-Cad: E-Cadherin
EMT: Epithelial mesenchymal transition
ESC: Embryonic stem cells
FN: Fibronectin
GEO: Gene Expression Omnibus
IHC: Immunohistochemistry
lncRNA: Long noncoding RNA
m^6^A: N6-methyladenosine
OS: Overall survival
qRT-PCR: Quantitative real-time PCR
RBP: RNA binding protein
RIP: RNA Immunoprecipitation
ROS: Reactive oxygen species
TCGA: The Cancer Genome Atlas
TF: Transcription factor
Vim: Vimentin
