# Supplementary material for: m6A-induced lncRNA RP11 triggers the dissemination of colorectal cancer cells via upregulation of Zeb1
Source: Mol Cancer. 2019 Apr 13;18:87. doi: 10.1186/s12943-019-1014-2 (PMC6461827; doi:10.1186/s12943-019-1014-2)
Supplement: Supplementary file 1 — Figure S1. RP11 is increased during the tumourigenesis and progression of CRC. Figure S2. RP11 triggers the dissemination of CRC cells both in vitro and in vivo. Figure S3. Upregulation of Zeb1 mediates RP11-triggered dissemination of CRC cells. Figure S4. Downregulation of Siah1 and Fbxo45 mediates RP11-induced upregulation of Zeb1. Figure S5. RP11 regulates Siah1 and Fbxo45 expression by forming the RP11-hnRNPA2B1-mRNA complex. Figure S6. The m6A modification is involved in the upregulation of RP11 in CRC cells. Figure S7. The m6A/RP11/Zeb1 axis and in vivo progression of CRC. (DOCX 14708 kb) [file 12943_2019_1014_MOESM1_ESM.docx]

**Supplementary data for**

**m^6^A-induced lncRNA RP11 triggers the dissemination of colorectal cancer cells via upregulation of Zeb1**

**
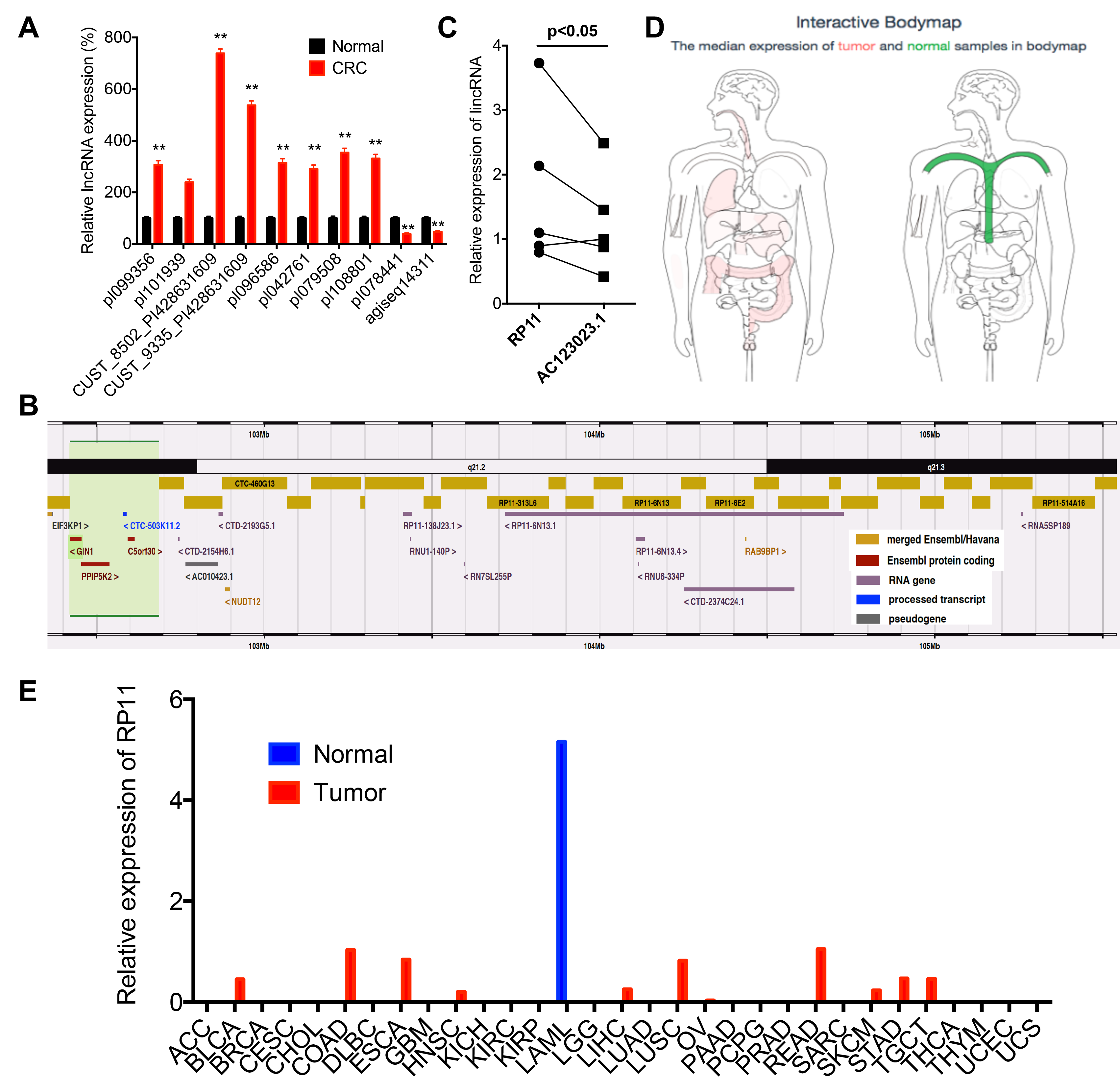
**

**Figure S1 RP11 is increased during the tumourigenesis and progression of CRC**

(A) The expression levels of 10 selected lncRNAs in 5 pairs of CRC and corresponding nontumour tissues were verified by qRT-PCR.

(B) The genomic locus of RP11 in human chr5. NUDT12, C5orf30, PPIP5K2, GIN1, RP11-6N13.1, and CTD-2374C24 are the adjacent transcripts.

(C) The expression of RP11 and lncRNA AC123023.1 in 5 CRC tissues was verified by qRT-PCR.

(D) The median expression of RP11 in tumour (*red*) and normal (*green*) samples in a body map.

(E) The gene expression profile of RP11 across all tumour samples (*red*) and paired normal tissues (*blue*) based on GEPIA. ACC, Adrenocortical carcinoma; BLCA, Bladder urothelial carcinoma; BRCA, Breast invasive carcinoma; CESC, Cervical squamous cell carcinoma and endocervical adenocarcinoma; CHOL, Cholangiocarcinoma; COAD, Colon adenocarcinoma; DLBC, Lymphoid neoplasm diffuse large B-cell lymphoma; ESC, Oesophageal carcinoma; GBM, Glioblastoma multiforme; HNSC, Head and neck squamous cell carcinoma; KICH, Kidney chromophobe; KIRC, Kidney renal clear cell carcinoma; KIRP, Kidney renal papillary cell carcinoma; LAML, Acute myeloid leukaemia; LGG, Low-grade glioma; LIHC, Liver hepatocellular carcinoma; LUAD, Lung adenocarcinoma; LUSC, Lung squamous cell carcinoma; OV, Ovarian serous cystadenocarcinoma; PAAD, Pancreatic adenocarcinoma; PCPG, Pheochromocytoma and paraganglioma; PRAD, Prostate adenocarcinoma; READ, Rectum adenocarcinoma; SARC, Sarcoma; SKCM, Skin cutaneous melanoma; STAD Stomach adenocarcinoma; TGCT, Testicular germ cell tumours; THCA, Thyroid carcinoma; THYM, Thymoma; UCEC, Uterine corpus endometrial carcinoma; UCS, Uterine carcinosarcoma.

Data are presented as the means ± SD from three independent experiments. *p<0.05, **p<0.01 compared with control.

Related to Figure 1


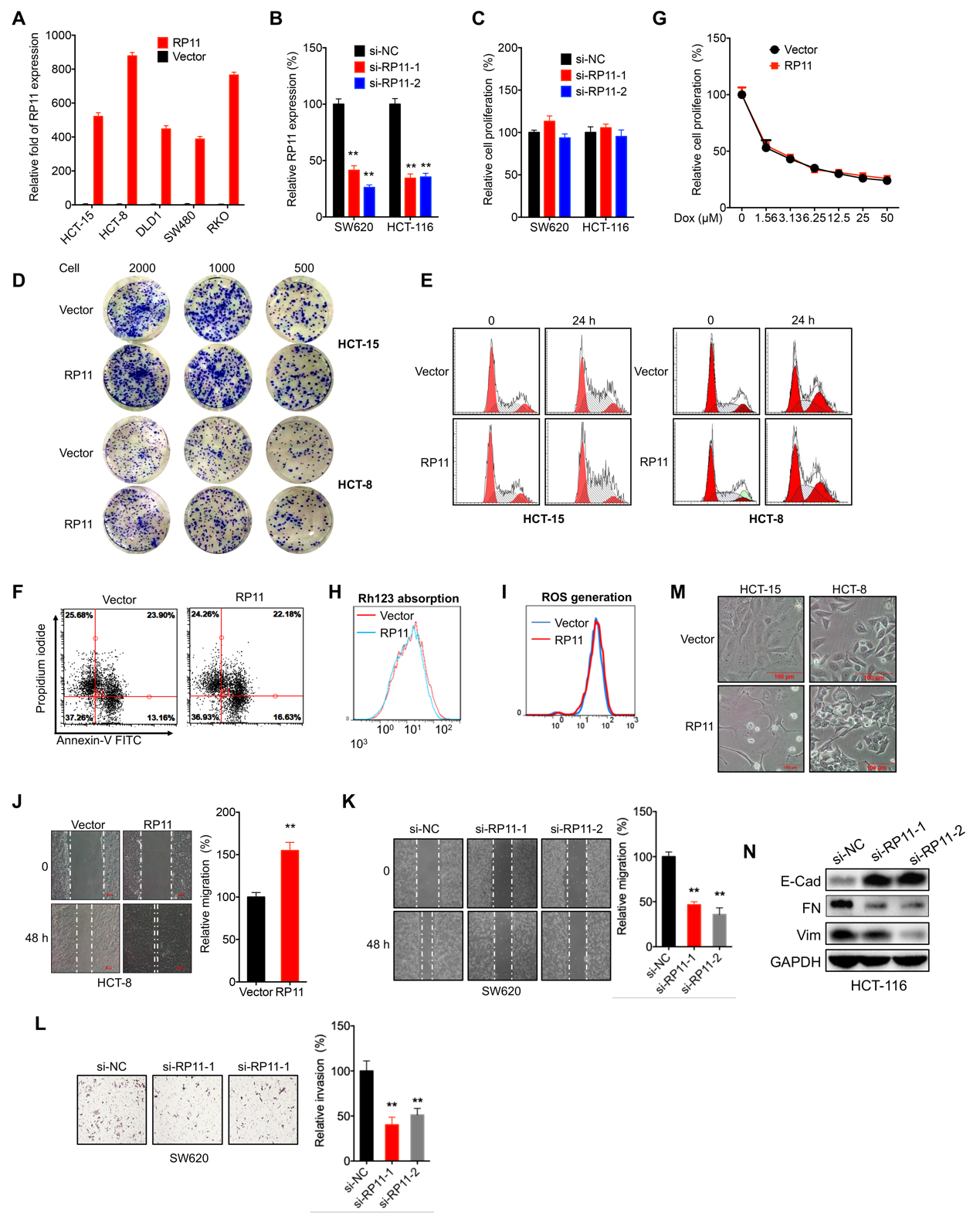


**Figure S2 RP11 triggers the dissemination of CRC cells both *in vitro* and *in vivo***

1. CRC cells were transfected with vector control or pcDNA/RP11 for 24 h, and RP11 expression was verified by qRT-PCR.
2. SW620 or HCT-116 cells were transfected with si-NC or si-RP11-1/-2 for 24 h, and RP11 expression was verified by qRT-PCR. si-RP11-2 was used for the subsequent experiments due to its better efficiency.
3. After transfection with si-NC or si-RP11-1/-2 for 48 h, the proliferation of SW620 and HCT-116 cells was measured with a CCK-8 kit.
4. HCT-15 or HCT-8 RP11 stable overexpression and control cells (2 × l0^5^) were cultured in 6-well plates for two weeks before colonies were counted.
5. HCT-15 or HCT-8 RP11 stable overexpression and control cells were synchronized at the G1/S transition by a double TdR block and then further cultured for 24 h. The cell cycles were analysed by FCM.
6. HCT-15 RP11 stable overexpression and control cells were treated with 100 μM NaAsO_2_ for 6 h, stained with annexin V-FITC and PI, and analysed by FCM for cell apoptosis.
7. HCT-15 RP11 stable overexpression and control cells were treated with increasing concentrations of Dox for 24 h, and cell proliferation was measured with a CCK-8 kit.
8. HCT-15 RP11 stable overexpression and control cells were treated with Rh123 (5 μM) for 2 h. Then, the cells were washed five times with cold PBS and digested, and fluorescence intensity was measured by FCM.
9. HCT-15 RP11 stable overexpression and control cells were treated with CM-H2DCFDA, and fluorescence intensity was measured by FCM.
10. The wound healing of HCT-8 RP11 stable overexpression and control cells was recorded (*left*) and quantitatively analysed (*right*).
11. After transfection with si-NC or si-RP11 for 48 h, the wound healing of SW620 cells was recorded (*left*) and quantitatively analysed (*right*).
12. The *in vitro* invasion of SW620 cells transfected with si-NC or si-RP11 for 48 h was recorded (*left*) and quantitatively analysed (*right*).
13. Phenotypic changes in HCT-15 or HCT-8 RP11 stable overexpression and control cells were detected using a phase contrast microscope.
14. After transfection with si-NC or si-RP11 for 48 h, the expression of EMT markers in HCT-116 cells was verified by western blot analysis.

Data are presented as the mean ± SD from three independent experiments. **p<0.01 compared with control.

Related to Figure 2


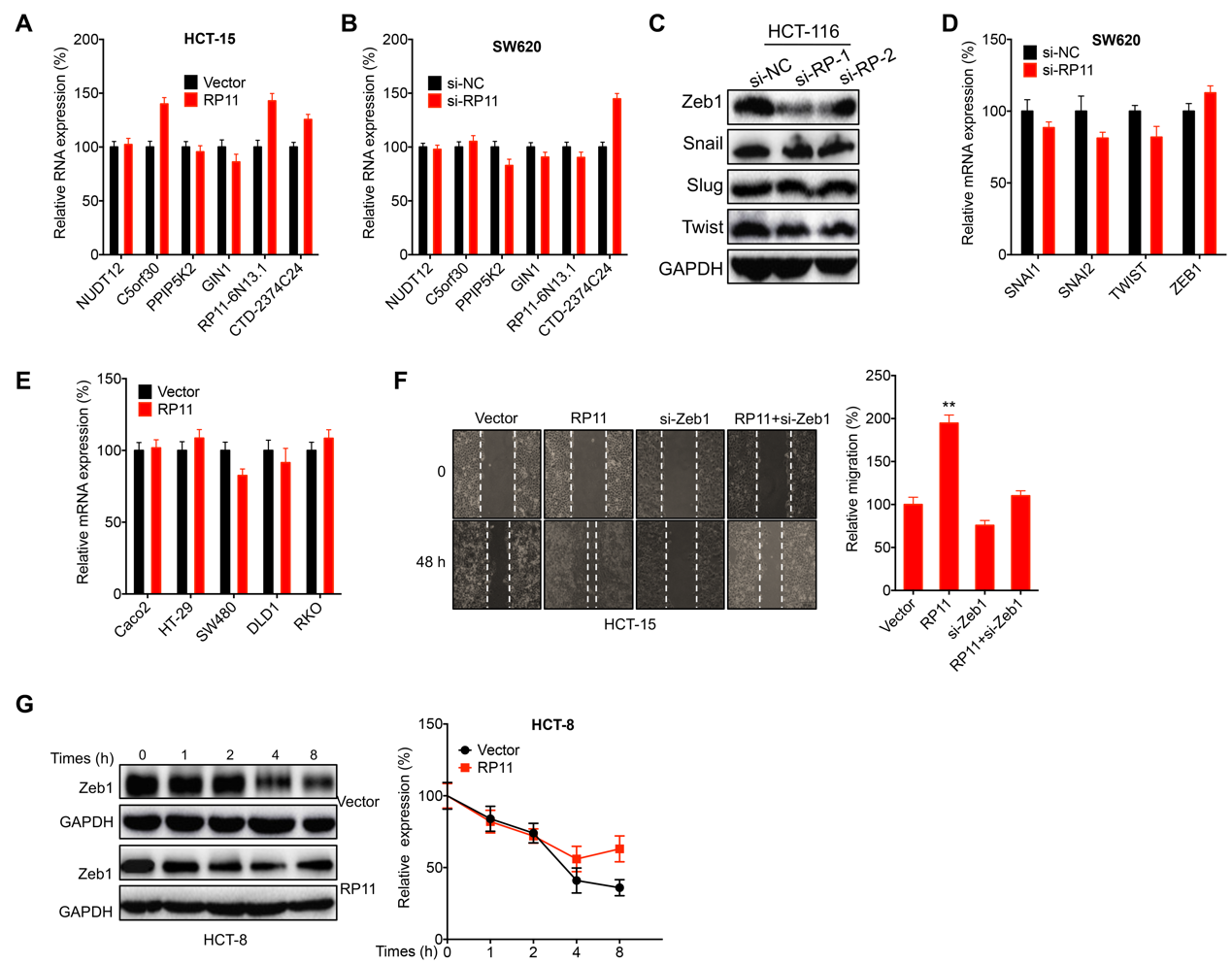


**Figure S3 Upregulation of Zeb1 mediates RP11-triggered dissemination of CRC cells**

1. The expression of adjacent transcripts in HCT-15 RP11 stable overexpression and control cells was verified by qRT-PCR.
2. After transfection with si-NC or si-RP11 for 24 h, the expression of RP11 adjacent transcripts in SW620 cells was verified by qRT-PCR.
3. After transfection with si-NC or si-RP11 for 48 h, the expression levels of EMT-TFs in HCT-116 cells were verified by western blot analysis.
4. After transfection with si-NC or si-RP11 for 24 h, the expression of EMT-TFs in SW620 cells was verified by qRT-PCR.
5. After transfection with vector or pcDNA/RP11 for 24 h, the mRNA expression of Zeb1 in CRC cells was verified by qRT-PCR.
6. After transfection with si-NC or si-ZEB1 for 24 h, the wound healing of HCT-15 RP11 stable overexpression and control cells was recorded (*left*) and quantitatively analysed (*right*).
7. After treatment with 100 μg/ml CHX for the indicated times, the expression of Zeb1 in HCT-8 RP11 stable overexpression and control cells was detected by western blot analysis (*left*) and quantitatively analysed (*right*).

Data are presented as the mean ± SD from three independent experiments.

Related to Figure 3.


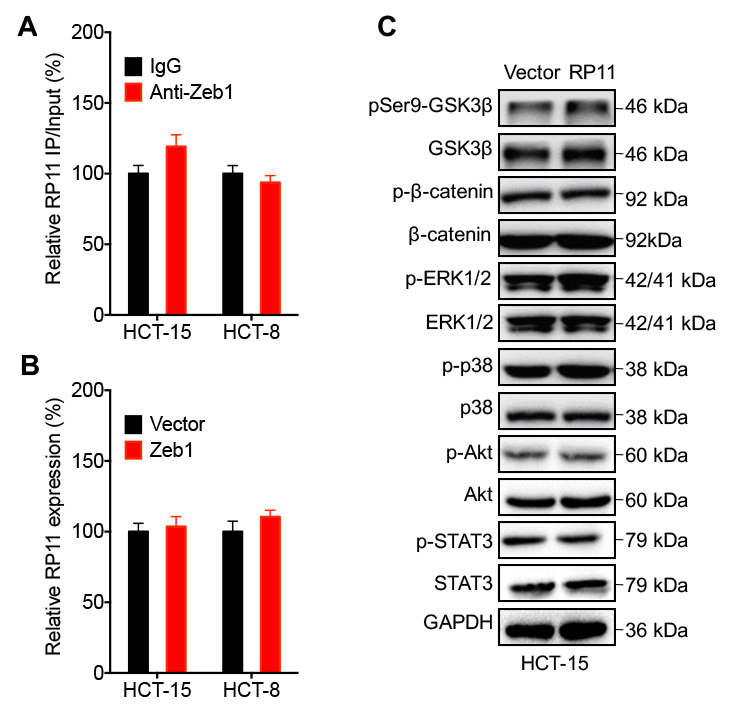


**Figure S4 Downregulation of Siah1 and Fbxo45 mediates RP11-induced upregulation of Zeb1**

(A) The relative enrichment of RP11 on endogenous Zeb1 in HCT-15 and HCT-8 cells was analysed by RIP-PCR with an IgG or anti-Zeb1 antibody.

(B) After transfection with vector control or pcDNA/Zeb1 for 24 h, RP11 expression was analysed by qRT-PCR.

(C) The total and phosphorylated levels of signalling molecules in HCT-15 RP11 stable overexpression and control cells were analysed by western blot.

Data are presented as the mean ± SD from three independent experiments.

Related to Figure 4.

**
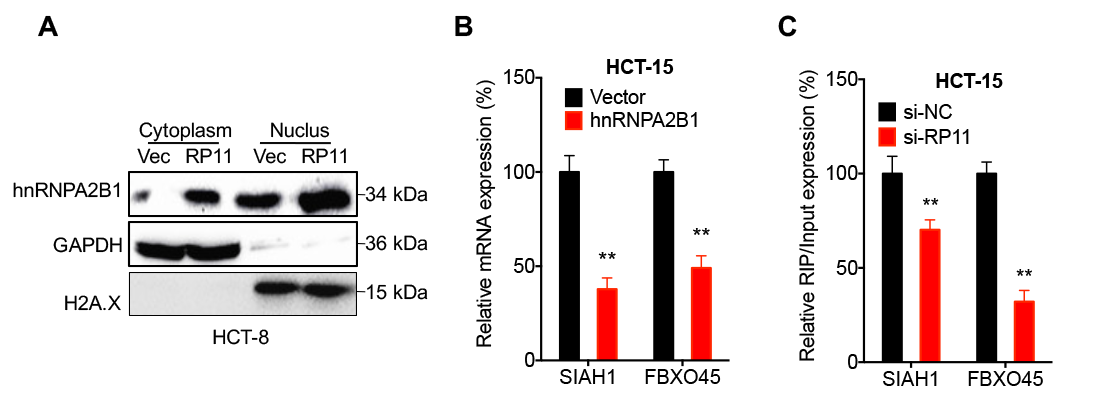
**

**Figure S5 RP11 regulates Siah1 and Fbxo45 expression by forming the RP11-hnRNPA2B1-mRNA complex**

(A) HnRNPA2B1 expression in the cytoplasmic and nuclear fractions of HCT-8 cells transfected with vector control or pcDNA/RP11 was analysed by western blot.

(B) After transfection with vector control or the hnRNPA2B1 construct for 24 h, the mRNA expression of Siah1 and Fbxo45 in HCT-15 cells was verified by qRT-PCR.

(C) Binding between hnRNPA2B1 and Siah1 mRNA or between hnRNPA2B1 and FBXO45 mRNA in HCT-15 cells transfected with si-NC or si-RP11 for 24 h was analysed by RIP-PCR with an anti-hnRNPA2B1 antibody.

Data are presented as the mean ± SD from three independent experiments. **p<0.01 compared with control.

Related to Figure 5


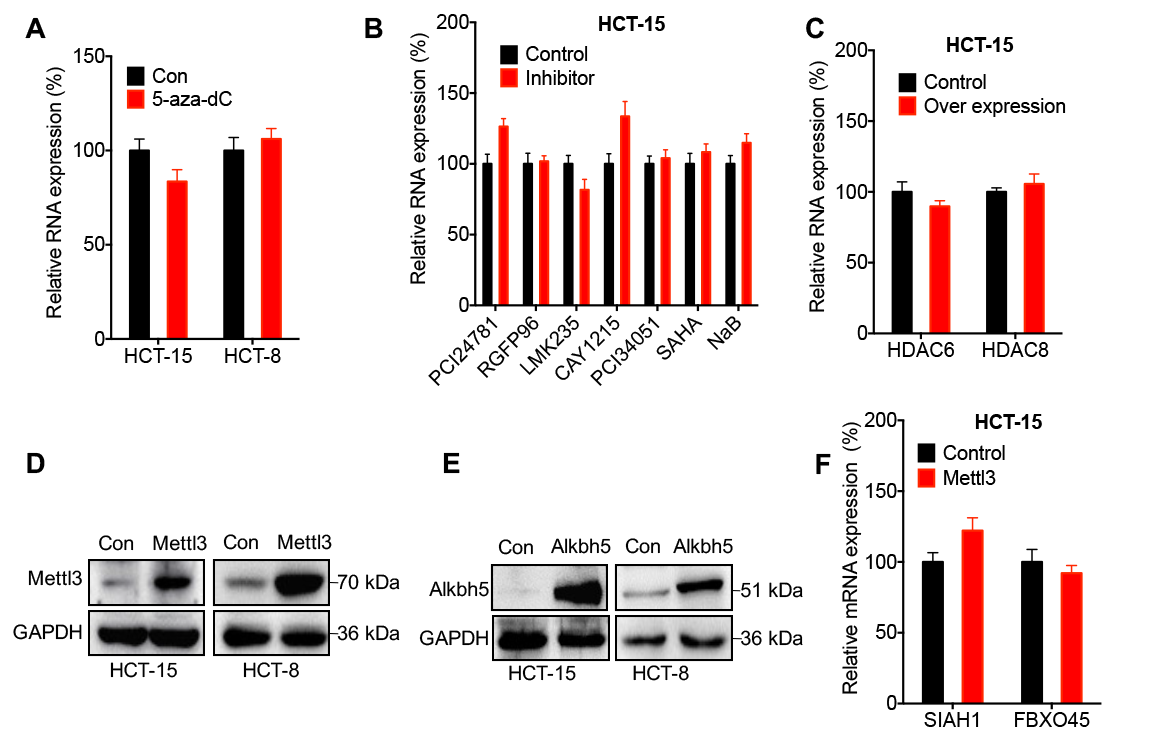


**Figure S6 The m^6^A modification is involved in the upregulation of RP11 in CRC cells**

1. HCT-15 or HCT-8 cells were treated with or without 5 µM 5-aza-dC for 4 days, and RP11 expression was measured by qRT-PCR.
2. HCT-15 cells were treated with specific inhibitors of HDAC1 (PCI24781, 8 µM), HDAC3 (GFRP96, 1 µM), HDAC4 (LMK235, 1 µM), HDAC6 (CAY1215, 4 µM), HDAC8 (PCI34051, 5 µM), SAHA (2 µM), or NaB (2 mM) for 24 h, and RP11 mRNA was measured.
3. After transfection with vector control, pcDNA/HDAC6, or pcDNA/HDAC8 for 24 h, RP11 expression in HCT-15 cells was measured by qRT-PCR.
4. After transfection with vector control or ppB/Mettl3 for 24 h, Mettl3 expression in HCT-15 and HCT-8 cells was verified by western blot analysis.
5. After transfection with vector control or pcDNA/Alkbh5 for 24 h, Alkbh5 expression in HCT-15 and HCT-8 cells was verified by western blot analysis.
6. After transfection with vector control or ppB/Mettl3 for 24 h, the mRNA levels of Siah1 and Fbxo5 in HCT-15 cells were measured by qRT-PCR.

Data are presented as the mean ± SD from three independent experiments.

Related to Figure 6

**Figure S7 The m^6^A/RP11/Zeb1 axis and *in vivo* progression of CRC**

(A&B) The relative mRNA expression levels of METTL3 (A) and Siah1 (B) in patients with stage N0, N1, and N2 CRC based on data available from TCGA database.

(C) The Pearson correlation between RP11 and ALKBH5 in 270 CRC tissues from TCGA database.

(D) OS of CRC patients with high (n=135) and low (n=134) levels of RP11 was plotted according to the Kaplan-Meier method.

(E) OS of CRC patients with high (n=135) and low (n=135) levels of RP11/Siah1 was plotted according to the Kaplan-Meier method.

(F) OS of CRC patients with high (n=135) and low (n=135) levels of RP11/FBXO45 was plotted according to the Kaplan-Meier method.

(G) OS of CRC patients with high (n=135) and low (n=134) levels of ZEB1 was plotted according to the Kaplan-Meier method.

(H) OS of CRC patients with high (n=135) and low (n=135) levels of ZEB1/Siah1 was plotted according to the Kaplan-Meier method.

(I) OS of CRC patients with high (n=135) and low (n=135) levels of ZEB1/FBXO45 was plotted according to the Kaplan-Meier method.

*p<0.05 compared with control.

Related to Figure 7

**MATERIALS AND METHODS**

**Patient sample collection**

Table S1 (Supplementary data) contains the detailed information of a group of 32 clinicopathologically characterized patients with histologically confirmed CRC from the Sun Yat-sen Memorial Hospital of Sun Yat-sen University collected between January 2014 and February 2015. All tissue samples were selected by an experienced pathologist immediately after surgical resection, snap frozen in liquid nitrogen, and stored at −80 °C. The expression of lncRNAs was measured by real-time PCR.

**Cell culture, transfection and stable cell line generation**

Human CRC cell lines SW620, LoVo, HCT-116, Caco2, HT29, HCT-15, HCT-8, SW480, DLD1, and RKO were obtained from American Type Culture Collection (Manassas, VA, USA). Human normal colon mucosal epithelial NCM460 cells were purchased from InCell (San Antonio, TX, USA). Cells were cultured in RPMI 1640 or DMEM with 5% foetal bovine serum at 37 °C in a 5% CO_2_ atmosphere. An ABI 3130 Genetic Analyzer (Applied Biosystems) was used for profiling. The DNA profile data were cross-checked with the ATCC data bank. Twenty-four hours before transfection, the medium was replaced with fresh medium and transfected using Lipofectamine 2000 reagent (Invitrogen) with vector control, plasmid construct, siRNA negative control (si-NC), or siRNAs according to the manufacturer’s instructions. The working concentration of siRNA was 50 nM.

To generate RP11 stable overexpression CRC cells, HCT-15 and HCT-8 cells transfected with the pcDNA3.1 or pcDNA/RP11 (containing full-length RP11) vector were selected with 1 μg/ml neomycin for two weeks. Control cell lines were generated similarly with the vector control. The RP11 stable overexpression and control cells were cultured in medium supplemented with 1 μg/ml neomycin. Cells were incubated with medium without neomycin for four days before experiments.

**RNA extraction and quantitative real-time PCR**

RNA extraction with Trizol (Invitrogen) and real-time PCR were performed according to the protocol used in our previous study [18]. Quantitative real-time PCR (qRT-PCR) was implemented with an iCycler (Bio-Rad, Hercules, USA) using validated primers and SYBR Premix Ex Taq II (Takara, Japan). The primers for the targeted genes are listed in Table S2. For quantitative RT-PCR, GAPDH was used as an endogenous control for cytoplasmic or total RNA, while U6 RNA was selected as an endogenous control for nuclear RNA. Expression levels were calculated using the 2^− ΔΔCt^ method. Three independent experiments were performed.

**Reagents**

All chemicals were purchased from Sigma Chemical Co. (St. Louis, MO, USA) unless otherwise noted. Monoclonal antibodies against fibronectin (FN), E-Cadherin (E-Cad), vimentin (Vim), Snail, Slug, Twist, Zeb1, histone H2A.X, ubiquitin, Siah1, Fbxo45, p-GSK-3β (Ser9), GSK-3β, p-β-catenin (Ser33/37/Thr41), β-catenin, p-ERK1/2(Thr202/Tyr204), ERK1/2, p-p38-MAPK (Thr180/Tyr182), p38-MAPK, p-STAT3 (Tyr 705), STAT3, hnRNPA2B1, Mettl3, Alkbh5, and GAPDH were from Cell Signaling Technology Inc. (Beverly, MA, USA). Antibodies against p-Akt (Ser473) and Akt were purchased from Bioworld Technology Inc. Horseradish peroxidase-conjugated secondary antibody was purchased from Santa Cruz Biotechnology (Santa Cruz, CA, USA). All compounds were dissolved in DMSO. Medium containing 0.5% DMSO was used as the control.

**Cell proliferation assay**

The effects of lncRNA on the proliferation of CRC cells were evaluated with the CCK-8 kit (Dojindo Molecular Technologies, Gaithersburg, MD, USA). All experiments were performed in triplicate.

***In vitro* wound healing assay and transwell assay**

Wound healing assays were performed to assess the migratory ability of CRC cells as previously described ^1^. The distance migrated was calculated by subtracting the average distance between wound edges from the distance at the beginning. Cell invasion assays were performed using 24-well transwell plates (8-μm pore, Corning) according to our previous study ^2^. Briefly, polycarbonate filters pre-coated with Matrigel Matrix (20 mg/ml, BD Biosciences) were used for the invasion assays. Cells (1×10^5^) in 300 μl medium (containing 0.1% FBS) were seeded in the upper chambers. Then, 600 μl medium with 10% FBS was added to the lower chambers and served as a chemotactic agent. After incubation for the indicated time periods, the number of penetrated cells was counted under a phase contrast microscope (five random fields per chamber). The invasion assays were carried out in at least 5 independent experi­ments.

**Western blot analysis**

Western blot analysis was performed as previously described ^3^. To measure the subcellular localization of Zeb1, cytoplasmic and nuclear extracts were prepared using NE-PER nuclear and cytosolic extraction reagents (Pierce). GAPDH and H2A.X were used as loading controls for the cytoplasm and nucleus, respectively.

***Immunohistochemistry (IHC)***

IHC was performed to measure the *in vivo* effect of RP11 on the expression of Ki67, vim, FN, Zeb1, Siah1, and Fbxo45 according to our previous study ^3^. Briefly, tumour tissues were fixed in formalin and embedded in paraffin. For immunohistochemical staining, sections were deparaffinized and hydrated, and endogenous peroxidase activity was blocked with 3% H_2_O_2_ in water for 10 min. Antigen retrieval was performed with 10 mM citrate buffer (pH 6.0) for 10 min. Slides were blocked with Biocare reagent for 10 min and then incubated with primary antibodies overnight at 4 °C. After two washes in PBS, slides were incubated with goat anti-rabbit horseradish peroxidase-conjugated secondary antibodies for 30 min at room temperature and then washed. Finally, slides were incubated with 3,3’-diamionobenzidine and counterstained with haematoxylin.

**Soft agar colony formation assay**

Cells were trypsinized into a single-cell suspension 48 h after transfection. For the soft agar colony formation assay, 2000 cells were seeded in each well of a 6-well plate that contained soft agar (Gibco, Grand Island, New York, USA) and then maintained for 2 weeks to allow colony formation. Colonies with diameters greater than 150 μm were counted.

**Analysis of fluorescence intensity by flow cytometry**

For visualization of the effects of RP11 on the intracellular retention of Rh123, HCT-15 RP11 stable overexpression or control cells were seeded onto 6-well-plate slides on the day prior to the assay. After exposure to 5 mM Rh123 for 2 h, the cells were washed three times with cold PBS. Fluorescence intensity was measured by flow cytometry using 488 nm excitation and 535 nm emission wavelengths.

To analyse the effects of RP11 on reactive oxygen species (ROS) generation, HCT-15 RP11 stable overexpression or control cells were seeded on poly-L-lysine-coated glass coverslips (8×8 mm) in 24-well plates and incubated for 20 min at 37 °C with 10^-5^ M DCF-DA in serum-free medium. Subsequently, cells were resuspended in 300 μl PBS before measurements were made. Fluorescence histograms were recorded with a Coulter Epics XL Flow Cytometry System (Beckman-Coulter, Miami, FL, USA) and analysed using Cell Quest software supplied by the manufacturer.

***Immunoprecipitation***

After lysis and centrifugation, input was performed with 2.5% of the crude lysate. Equal amounts of Zeb1 were immunoprecipitated with a preclearing process and incubated overnight at 4 °C with the ubiquitin antibody and protein A/G as indicated (SCBT, sc-2003). As a control, immunoprecipitation with rabbit IgG (SCBT, sc-11390) was also conducted. After 4 washes with lysis buffer and one wash with PBS plus inhibitors, pellets were resuspended in 6× loading buffer, boiled and loaded onto 8% polyacrylamide gels and transferred to PVDF membranes (Immobilon-P, Millipore). The ubiquitylation of Zeb1 was measured by western blot analysis.

**References**

1. Chen ZJ, Yang XL, Liu H, Wei W, Zhang KS, Huang HB*, et al.* Bisphenol A modulates colorectal cancer protein profile and promotes the metastasis via induction of epithelial to mesenchymal transitions. *Arch Toxicol* 2015, **89**(8)**:** 1371-1381.

2. Chen ZJ, Wei W, Jiang GM, Liu H, Wei WD, Yang X*, et al.* Activation of GPER suppresses epithelial mesenchymal transition of triple negative breast cancer cells via NF-kappaB signals. *Mol Oncol* 2016, **10**(6)**:** 775-788.

3. Liu Q, Chen ZJ, Jiang GM, Zhou Y, Yang XL, Huang HB*, et al.* Epigenetic down regulation of G protein-coupled estrogen receptor (GPER) functions as a tumor suppressor in colorectal cancer. *Molecular Cancer* 2017, **16**.
