# Supplementary material for: m6A-induced lncRNA RP11 triggers the dissemination of colorectal cancer cells via upregulation of Zeb1
Source: Mol Cancer. 2019 Apr 13;18:87. doi: 10.1186/s12943-019-1014-2 (PMC6461827; doi:10.1186/s12943-019-1014-2)
Supplement: Supplementary file 2 — Table S1. The clinic pathological features of clinical CRC tissues (n = 32). Table S2. Sequences of primers. Table S3. The information of 8 lncRNAs. Table S4. The protein information of RP11 pull down/MS analysis. Table S5. Factors related to the stability of Zeb1 in cancer cells. (ZIP 279 kb) [file 12943_2019_1014_MOESM2_ESM.zip › 12943_2019_1014_MOESM2_ESM/Table S1.docx]

**Table S1 The clinic pathological features of clinical CRC tissues (n=32)**

| **Characteristics** | | **N** | **Expression levels** | **p value** |
| --- | --- | --- | --- | --- |
| Tumor/Adjacent | Tumor | 32 | 63.8 ± 70.6 | <0.01 |
|  | Adjacent | 32 | 1.32 ± 2.59 |  |
| Age | ≤50 | 11 | 55.5 ± 70.8 | 0.913 |
|  | ＞50 | 21 | 52.3 ± 36.1 |  |
| Sex | Male | 11 | 69.5 ± 49.1 | 0.518 |
|  | Female | 21 | 56.2 ± 40.3 |  |
| Stage | 1 | 4 | 43.0 ± 29.0 | 0.116 |
|  | 2 | 14 | 51.7 ± 43.7 |  |
|  | 3 | 10 | 55.5 ± 37.6 |  |
|  | 4 | 4 | 64.9 ± 66.2 |  |
