# Supplementary material for: m6A-induced lncRNA RP11 triggers the dissemination of colorectal cancer cells via upregulation of Zeb1
Source: Mol Cancer. 2019 Apr 13;18:87. doi: 10.1186/s12943-019-1014-2 (PMC6461827; doi:10.1186/s12943-019-1014-2)
Supplement: Supplementary file 2 — Table S1. The clinic pathological features of clinical CRC tissues (n = 32). Table S2. Sequences of primers. Table S3. The information of 8 lncRNAs. Table S4. The protein information of RP11 pull down/MS analysis. Table S5. Factors related to the stability of Zeb1 in cancer cells. (ZIP 279 kb) [file 12943_2019_1014_MOESM2_ESM.zip › 12943_2019_1014_MOESM2_ESM/Table S2 Sequences of primers.docx]

**Table S2 Sequences of primers**

| Name |  | Sequences |
| --- | --- | --- |
| GAPDH | Sense | 5'- GTCTCCTCTGACTTCAACAGCG-3' |
|  | Anti-sense | 5'- ACCACCCTGTTGCTGTAGCCAA-3' |
| agiseq32100 | Sense | 5'- TTAGGTCGGCTCCGTATGAC -3' |
|  | Anti-sense | 5'- GGAGGATCACGTGGGATGAT-3' |
| agiseq31710 | Sense | 5'- CCTGCAATTCCTTGCCACTT -3' |
|  | Anti-sense | 5'- ATGTCCATGATTGCCTGCAC -3' |
| CUST_4882_PI428631609 | Sense | 5'- GAAGGCTGGAGTTGCTTGAG -3' |
|  | Anti-sense | 5'- ACCCTTGTCCAAACATGCAC -3' |
| agiseq49024 | Sense | 5'- GAACCAGGTGGCCCTAGATT -3' |
|  | Anti-sense | 5'- AACGGAGGAACGGTATTGGT -3' |
| pI078441 | Sense | 5'- GCAGCCCTGTCCATTGTATG -3' |
|  | Anti-sense | 5'- TGACAGTAGGAAGCAGCACT -3' |
| agiseq14311 | Sense | 5'- AGATCCTGGGATGAGGAGGT -3' |
|  | Anti-sense | 5'- AGAGCCAGGAGGATTTGGAC -3' |
| Pre-RP11 | Sense | 5'- CATTCCTGTGGGTTCTTGGT -3' |
|  | Anti-sense | 5'- CATCTGAAGGAACAAACTCCGGA-3' |
| RP11(CUST_8502_PI428631609) | Sense | 5'- CATTCCTGTGGGTTCTTGGT -3' |
|  | Anti-sense | 5'- GAAGTACGGCACCAGCATTT -3' |
| SNAI1 | Sense | 5'- TGCCCTCAAGATGCACATCCG A-3' |
|  | Anti-sense | 5'- GGGACAGGAGAAGGGCTTCTC -3' |
| SNAI2 | Sense | 5'-ATCTGCGGCAAGGCGTTTTCCA -3' |
|  | Anti-sense | 5'- GAGCCCTCAGATTTGACCTGTC- 3' |
| TWIST | Sense | 5'- GCCAGGTACATCGACTTCCTCT -3' |
|  | Anti-sense | 5'- TCCATCCTCCAGACCGAGAAGG -3' |
| ZEB1 | Sense | 5'- GGCATACACCTACTCAACTACGG -3' |
|  | Anti-sense | 5'- TGGGCGGTGTAGAATCAGAGT C -3' |
| NUDT12 | Sense | 5'- GAGGAAGATGGATTGGTTGC -3' |
|  | Anti-sense | 5'- GCTGGCATAGGAGGATGAAG -3' |
| C5orf30 | Sense | 5'- CTACCAGCCATACGAGATTCC -3' |
|  | Anti-sense | 5'- AAGAAGACAAAGAGGCAGAGG- 3' |
| PPIP5K2 | Sense | 5'- CAGACCTGTGCGTCAGCTAAT -3' |
|  | Anti-sense | 5'- ATCTGCATGGTGGAAGAAATG-3' |
| GIN1 | Sense | 5'- ACTGCCAAGTGAGAGAAGTGG-3' |
|  | Anti-sense | 5'- AGGTGCTGTTTCGGTGCTAC- 3' |
| RP11-6N13.1 | Sense | 5'- TATGCCTAGTCCAGCCACAG-3' |
|  | Anti-sense | 5'- GAGCGAACAAAGCGGTGTAT-3' |
| CTD-2374C24 | Sense | 5'- CACCGTGGGATCTGAGAGTA-3' |
|  | Anti-sense | 5'- CACGAGGTCTTGCAAGGATG-3' |
| ATM | Sense | 5'- AGAGACAGGGTTGCCATTG -3' |
|  | Anti-sense | 5'- TGCAGAAAGAGTTCCAGCTT -3' |
| CSN5 | Sense | 5'- AGCGAGGTAAAGTTGCGTCT -3' |
|  | Anti-sense | 5'- AGGTTTTCTGGGCCATACCG -3' |
| FLASH | Sense | 5'-CTCTGAATAGTCCAGTGAGACCT-3' |
|  | Anti-sense | 5'-CTGACTCTTAGAGGTAGAATGAGC-3' |
| SENP1 | Sense | 5'- CAGCAGATGAATGGAAGTGA -3' |
|  | Anti-sense | 5'- CCGGAAGTATGGCATGTGT -3' |
| USP51 | Sense | 5'-AAAAGAATGCTTTAGGTGGG-3' |
|  | Anti-sense | 5'-AAGGAATACTCCCTGACTTC-3' |
| FBXO45 | Sense | 5'- AGAGGCTCTGCGCACGGACAT -3' |
|  | Anti-sense | 5'- TGTAGACATTCCTGGAGCAGTCA- 3' |
| SIAH1 | Sense | 5'- TCTTCCTGGTGCTGTTGACTGG-3' |
|  | Anti-sense | 5'- CGATTGCGAAGAACTGCTGGTG-3' |
| U6 | Sense | 5'- GGAACGATACAGAGAAGATTAGC-3' |
|  | Anti-sense | 5'- TGGAACGCTTCACGAATTTGCG -3' |
