# Supplementary material for: m6A-induced lncRNA RP11 triggers the dissemination of colorectal cancer cells via upregulation of Zeb1
Source: Mol Cancer. 2019 Apr 13;18:87. doi: 10.1186/s12943-019-1014-2 (PMC6461827; doi:10.1186/s12943-019-1014-2)
Supplement: Supplementary file 2 — Table S1. The clinic pathological features of clinical CRC tissues (n = 32). Table S2. Sequences of primers. Table S3. The information of 8 lncRNAs. Table S4. The protein information of RP11 pull down/MS analysis. Table S5. Factors related to the stability of Zeb1 in cancer cells. (ZIP 279 kb) [file 12943_2019_1014_MOESM2_ESM.zip › 12943_2019_1014_MOESM2_ESM/Table S5 .docx]

**Table S5 Factors related to the stability of Zeb1 in cancer cells**

| **Factors** | **Effect** | **Mechanisms** | **Ref** |
| --- | --- | --- | --- |
| ATM | Stabilization | ATM can stabilize ZEB1 through phosphorylating it at S585 | [1] |
| CSN5 | Stabilization | CSN5 directly interacts with ZEB1 and inhibits its ubiquitination and stabilizes ZEB1 | [2] |
| FLASH | Stabilization | FLASH might conceal the UBL recognition sites on ZEB1 or compete with the ZEB1 UBLs for binding through its direct interaction with ZEB1. | [3] |
| SENP1 | Stabilization | Senp1 silencing decrease Zeb1 protein level in HCC cells | [4] |
| USP51 | Stabilization | USP51 as a deubiquitinase that binds, deubiquitinates, and stabilizes ZEB1 | [5] |
| Fbxo45 | Degradation | Fbxo45, as a module of SPFFbxo45 complex, induces the degradation of EMT-TFs including Zeb1 and Zeb2. | [6] |
| SIAH1 | Degradation | Siah1 interacts with and target Zeb1 for proteasomal degradation | [7] |

**References**

1 Zhang P, Wei Y, Wang L, Debeb BG, Yuan Y, Zhang J*, et al.* ATM-mediated stabilization of ZEB1 promotes DNA damage response and radioresistance through CHK1. Nat Cell Biol 2014;**16**:864-75.

2 Zhang S, Hong Z, Chai Y, Liu Z, Du Y, Li Q*, et al.* CSN5 promotes renal cell carcinoma metastasis and EMT by inhibiting ZEB1 degradation. Biochem Biophys Res Commun 2017;**488**:101-8.

3 Abshire CF, Carroll JL, Dragoi AM. FLASH protects ZEB1 from degradation and supports cancer cells' epithelial-to-mesenchymal transition. Oncogenesis 2016;**5**:e254.

4 Zhang W, Sun H, Shi X, Wang H, Cui C, Xiao F*, et al.* SENP1 regulates hepatocyte growth factor-induced migration and epithelial-mesenchymal transition of hepatocellular carcinoma. Tumour Biol 2016;**37**:7741-8.

5 Zhou Z, Zhang P, Hu X, Kim J, Yao F, Xiao Z*, et al.* USP51 promotes deubiquitination and stabilization of ZEB1. Am J Cancer Res 2017;**7**:2020-31.

6 Xu M, Zhu C, Zhao X, Chen C, Zhang H, Yuan H*, et al.* Atypical ubiquitin E3 ligase complex Skp1-Pam-Fbxo45 controls the core epithelial-to-mesenchymal transition-inducing transcription factors. Oncotarget 2015;**6**:979-94.

7 Chen A, Wong CSF, Liu MCP, House CM, Sceneay J, Bowtell DD*, et al.* The ubiquitin ligase Siah is a novel regulator of Zeb1 in breast cancer. Oncotarget 2015;**6**:862-73.
